# A Preliminary Insight into Under-Researched Plants from the Asteraceae Family in the Balkan Peninsula: Bioactive Compound Diversity and Antioxidant Potential

**DOI:** 10.3390/plants14182904

**Published:** 2025-09-18

**Authors:** Sanja Vojvodić, Danica Božović, Milica Aćimović, Uroš Gašić, Zoran Zeković, Anica Bebek Markovinović, Danijela Bursać Kovačević, Bojan Zlatković, Branimir Pavlić

**Affiliations:** 1 Faculty of Technology Novi Sad, University of Novi Sad, Blvd. cara Lazara 1, 21000 Novi Sad, Serbia; sanjamilosevic9898@gmail.com (S.V.); danica_bozovic@live.com (D.B.); zzekovic@tf.uns.ac.rs (Z.Z.); 2 Institute of Field and Vegetable Crops Novi Sad, National Institute of Republic of Serbia, Maksima Gorkog 30, 21000 Novi Sad, Serbia; milica.acimovic@ifvcns.ns.ac.rs; 3 Department of Plant Physiology, Institute for Biological Research “Siniša Stanković”, National Institute of Republic of Serbia, University of Belgrade, Bulevar Despota Stefana 142, 11108 Belgrade, Serbia; uros.gasic@ibiss.bg.ac.rs; 4 Faculty of Food Technology and Biotechnology, University of Zagreb, Pierottijeva 6, 10000 Zagreb, Croatia; anica.bebek.markovinovic@pbf.unizg.hr (A.B.M.); danijela.bursac.kovacevic@pbf.unizg.hr (D.B.K.); 5 Department of Biology and Ecology, Faculty of Sciences and Mathematics, University of Niš, Višegradska 33, 18000 Niš, Serbia; bojanzlat@yahoo.com

**Keywords:** under-researched plants, Asteraceae family, bioactive compound diversity, antioxidant activity, LC-MS chemical profile

## Abstract

Natural resources rich in polyphenols from plants belonging to the Asteraceae family remain largely unexplored. The main goal of this study was to characterize under-studied Asteraceae plants in terms of different bioactive compounds, antioxidant potential, and chemical profile. Twenty-three samples from 19 plant species were analyzed using conventional solid/liquid extraction, and the contents of total phenolics (TP), flavonoids (TF), flavonols (FL), hydroxycinnamic acids (HCA) and condensed tannins (CT), as well as extraction yield were determined. Antioxidant activity was assessed using DPPH, ABTS and FRAP assays, and five plant samples were subjected to LC-MS analysis. Extraction yields ranged from 0.57% to 1.74%. *Solidago virgaurea* had the highest TP and FL contents, while *Tanacetum vulgare* showed the highest TF and HCA levels. The highest CT content was found in the roots of *Helianthus tuberosus*. Asteraceae species such as *S. virgaurea*, *Tussilago farfara*, *Cota tinctoria*, *T. vulgare*, and *Inula ensifolia* demonstrated the greatest antioxidant potential, with about 130 different identified compounds. Given the promising chemical richness of these under-researched species, future studies should focus on enhancing extraction of bioactive compounds using novel techniques and incorporating extracts as natural, non-synthetic preservatives in various products to improve their nutritional and biological properties.

## 1. Introduction

The rapid growth of the human population and its increasing demands for improved food and healthcare products are driving the need for new strategies to expand available natural resources. Simultaneously, their ongoing depletion and the rise in antibiotic resistance are accelerating the search for alternative solutions. In this context, the investigation of under-researched plant species, industrial by-products, and agricultural waste initiated in the current era should not only continue but also intensify. The vast diversity of untapped wild-plant sources offers a promising foundation for scientific exploration. From nature’s biodiversity, through extensive research, to sustainable commercialization, there is a significant scope for further innovation. Therefore, harnessing underutilized plants for household food, pharmaceuticals, and industrial applications holds significant potential, particularly when approached through environmentally sustainable practices [1,2].

The Asteraceae family, also known as the composite or sunflower family, is widespread, comprising approximately 25,000 species across 1600 genera [3]. The geographical regions richest in Asteraceae plants are the Mediterranean, Eastern Europe and Asia, particularly in arid and semi-arid zones [3]. Plants from this family are recognizable by their alternately arranged leaves and clustered inflorescences at the tops of their stems. Their flowers come in a variety of colors, making many species popular ornamental garden plants, while others are valued for their medicinal properties [4]. The most famous species include yarrow (*Achillea millefolium* L.), chamomile (*Matricaria chamomilla* L.), wormwood (*Artemisia absinthium* L.), artichoke (*Cynara scolymus* L.), dandelion (*Taraxacum officinale* L.), lettuce (*Lactuca sativa* L.) and sunflower *(Helianthus annuus* L.). This economically important family provides a wide range of products, including food (e.g., edible oil, leafy vegetables, sunflower seeds), ornamental plants (e.g., marigold (*Calendula officinalis*), chrysanthemum (*Chrysanthemum*), gerbera (*Gerbera jamesoni*)) and medicinal herbs (e.g., yarrow, wormwood, chamomile) [5] (Figure S1). Despite their diversity, members of the family share a common trait—they are a rich source of inulin, a natural polysaccharide with strong prebiotic properties [3]. Other notable secondary metabolites found in plants include phenolic acids (chlorogenic, caffeic, ferulic, *p*-coumaric), flavonoids (kaempferol, luteolin, quercetin, apigenin), sesquiterpene lactones (responsible for the plants’ bitter taste), as well as alkaloids, tannins, saponins, lignans, essential oils, coumarins, and carotenoids (A, B, C, E) [6]. Owing to this rich phytochemical composition, Asteraceae plants exhibit strong pharmacological activities including anti-inflammatory, antimicrobial, antioxidant, astringent, hepatoprotective, and diuretic effects [4]. Traditionally, these plants have been used to relax smooth muscles and to treat wounds, bleeding, headaches, pain, flatulence, dyspepsia, dysentery, lumbago and hemorrhoids [4].

In the flora of the Balkan Peninsula, the Asteraceae is the most widespread family in terms of the number of genera and species [7]. The family has a strong historical presence in the Peninsula, with over 80 species traditionally used as edible wild plants by the local population [8,9]. Among the most commonly consumed wild species are dandelion, chicory (*Cichorium intybus*), and yarrow [10]. Many species have been recorded as having edible roots (e.g., dandelion, chicory, inula), edible flowers (e.g., marigold (*Calendula officinalis*), safflower, blue cornflower (*Centaurea cyanus*), lily of the valley (*Convallaria majalis*)), and applications in herbal infusions or hot drinks (e.g., chamomile, yarrow, common wormwood, immortelle (*Helichrysum italicum*)) [11]. Conversely, a large number of medicinal plants within the family remain under-studied and scientifically overlooked.

Due to its immense diversity, the Asteraceae family holds vast untapped potential for producing extracts rich in bioactive compounds. Investigating their bioactivity and exploring their applications in the pharmaceutical industry for drug development or in the food industry to enhance the functional properties of food is both necessary and promising.

The main goal of this preliminary research was to characterize under-researched plants from the Asteraceae family native to the Balkan Peninsula, focusing on their content of various bioactive compound classes, antioxidant potential, and overall chemical profiles. The additional aim was to prioritize the most promising species for further, more advanced investigations.

## 2. Results and Discussion

### 2.1. Extraction Yield

**Figure 1:**
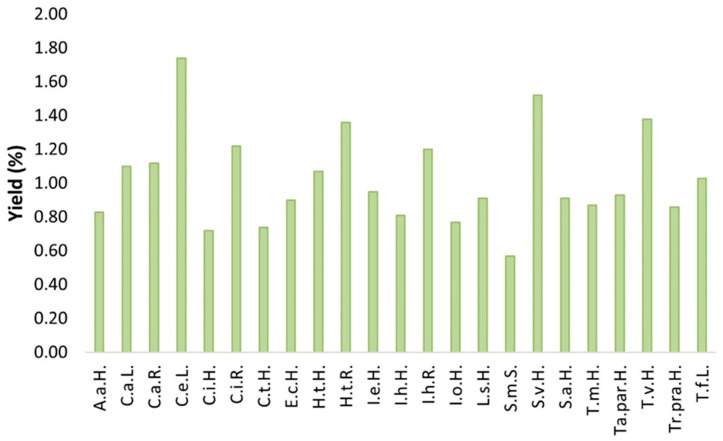
Extraction yield (EY) for different species from the Asteraceae.

The obtained extraction yield (EY) ranged from 0.57% to 1.74% across 23 species (Figure 1). The leaves of *Cichorium endivia* yielded an EY (1.74%) three times higher than the lowest EY obtained from *Silybum marianum* seeds (0.57%). The herbs of *Solidago virgaurea* and *Tanacetum vulgare*, as well as the root of *Helianthus tuberosus*, also provided highly satisfactory EYs—1.52%, 1.38%, and 1.36%, respectively.

Due to the largely unexplored potential of these species, very scarce literature data exists with few or no reports available regarding extraction yields. Existing scientific studies have primarily focused on the use of organic solvents—mainly methanol, acetone and hexane or water—in a solid/liquid extraction procedure. Consequently, direct comparison with the ethanol extracts obtained in this study is not feasible. However, the ethanol extracts from chamomile [12] and dandelion [13], as the main representatives of Asteraceae family, gave a higher yield in comparison to selected plants in this research. The main reasons for the differences in the obtained yields may be due to the choice of the appropriate solvent, solvent’s concentrations and also the chemical profile of mentioned species.

The obtained ethanol extracts provide an opportunity for safer and more applicable commercialization. These results enable better insight into the extraction yield of selected species of the Asteraceae family. According to the low amount of yields, we observed that there is room for green solvents, such as ethanol, to be used in conjunction with more advanced techniques to increase these yields.

### 2.2. Classes and Subclasses of Bioactive Compounds

#### 2.2.1. Total Phenolic (TP) and Flavonoid (TF) Content in Asteraceae Plant’s Extracts

**Figure 2:**
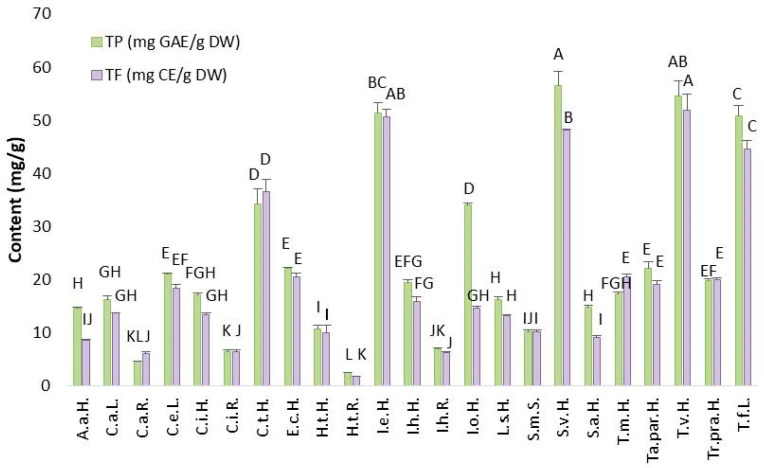
Total phenolic (TP) and flavonoid (TF) contents in Asteraceae plant extracts. Results are expressed as mean ± standard deviation (SD), and different letters indicate statistically significant differences (*p* ≤ 0.05) among the different plant extracts.

Total phenolic (TP) content, representing the major class of polar bioactive compounds, in the 23 species ranged from 2.45 ± 0.11 to 56.52 ± 2.72 mg GAE/g DW (Figure 2). Total flavonoids (TF), as a subgroup of phenolic compounds, were present in the range of 1.83 ± 0.05 to 51.85 ± 2.98 mg CE/g DW (Figure 2). The roots of *H. tuberosus* had the lowest content of both TP (2.45 ± 0.11 mg GAE/g DW) and TF (1.83 ± 0.05 mg CE/g DW), as well as its herb. Similarly, the roots of *Inula helenium, Cichorium inthybus*, *Carlina acanthifolia utzka* and the seeds of *S. marianum* exhibited low levels of both TP and TF. In general, the chemical profiles of aerial part and root in the plants differs. The aerial part mostly contains a higher content of TP and TF than roots, which was the reason for the lowest content of TP and TF in roots of selected species. On the other hand, extracts of E.c.H., C.i.H., Ta.par.H, Tr.pra.H., C.e.L., I.h.H., L.s.H., A.a.H., S.a.H., I.o.H., T.m.H and C.a.L. showed moderate amounts of TP and TF. The highest TP and TF contents were observed in five plants: S.v.H., T.v.H., T.f.L., C.t.H., and I.e.H. Among them, *S. virgaurea* had the highest TP content (56.52 ± 2.72 mg GAE/g DW), while *T. vulgare* had the highest TF content (51.85 ± 2.98 mg CE/g DW). Based on the contents of two major classes of biologically active compounds from which the antioxidant potential originates from, the selection of the species with the highest potential can be accomplished. Statistically significant differences in terms of TP and TF were observed in five plants (S.v.H., T.v.H., I.e.H., T.f.L., and C.t.H.) within 23 samples. Therefore, these species are chosen as the most promising candidates for further production of extracts and commercialization as non-synthetic preservatives and they are extensively discussed below.

A clearer understanding of the proportion of TF within TP is significant for further targeting the specific classes of bioactive compounds. A lower proportion of TF relative to TP was observed in A.a.H., S.a.H. and I.o.H., while moderate levels were found in S.v.H., T.f.L., C.i.H., Ta.par.H., C.e.L., I.h.H., L.s.H., C.a.L., I.h.R., and H.t.R. Considerably high TF content within the TP content was found in T.v.H., I.e.H., E.c.H., H.t.H., S.m.S., and C.i.R. Notably, some samples—C.t.H., Tr.pra.H., T.m.H., C.a.R.—exhibited higher TF than TP content, likely due to additional interferences detected during spectrophotometric analysis. Different levels of TF in TP are linked to the plant species chemical profile (some species contained more phenolic acids or tannins than flavonoids), a part of the plant (roots contained lower amount of flavonoids in comparison to leaves or flowers) [14], extraction method (not optimized for flavonoids) [15], environmental factors (plants gown under stress conditions will produce more flavonoids as defense mechanism) [16], and measurement issues [17].

The biological potential of two Asteraceae species (*Achillea biebersteinii* and *Anthemis tinctoria*) was investigated by Güçlü et al. [18]. Using 80% ethanol extracts, they measured TP and TF contents, along with antioxidant activity (via DPPH and ABTS assays). *Anthemis tinctoria* had higher TP and TF levels (94.9 ± 3.8 mg GAE/g and 29.9 ± 1.8 mg QE/g) in comparison to *Achillea biebersteinii* (65.5 ± 14.2 mg GAE/g and 20.5 ± 6.3 mg QE/g). In our study, the highest TP content observed among the tested plants was lower than that reported by Güçlü et al. [18], while the highest TF content exceeded theirs. Geographical climate, environmental factors and the time of plant harvesting can significantly affect the variation in TP content.

Furthermore, five other Asteraceae plants were evaluated for TP content [19]. Traditional solid/liquid extraction was performed using a 60:40 (*V*/*V*) methanol-water mixture for 3 h at 45 °C on *Achillea millefolium* L., *Arnica montana* L., *Calendula officinalis* L., *Chamaemelum nobile* L. and *Taraxacum officinale* L. Among them, *Arnica montana* showed the highest TP content, with a concentration of 119 mg/mL. Differences in TP content may be influenced by the choice of extraction solvent and growing conditions such as geographic place of the production, ripening stage, harvesting time and storage after harvesting [20]. These same variables may also explain the TP levels observed in our study.

The kinetic modeling of traditional solid/liquid extraction for isolating biologically active components from Asteraceae family plants was monitored in work of Tušek et al. [21]. Four well-known species were analyzed: *Matricaria recutita* L., *Taraxacum officinale*, *Calendula officinalis* and *Achillea millefolium* L. Four extraction parameters were varied: temperature, particle size of the material, extraction duration, and magnetic stirrer rotational speed. The highest temperature applied (80 °C) proved to be the most effective in terms of extraction yield, TP content, and antioxidant activity. The extract of *Achillea millefolium* obtained at 80 °C contained 40 mg GAE/g DM, followed by *Matricaria recutita* and *Taraxacum officinale.* The lowest TP content was found in *Calendula officinalis*. The TP content of the 23 species was of the same order of magnitude. Based on this comparison, we can conclude that by applying the same extraction techniques with similar parameters the content of TP in different Asteraceae species varied in a very narrow range. It can be highlighted that temperature, besides other extraction parameters, is the parameter that highly affected TP content variations in different Asteraceae species.

Scientific studies regarding the TP and TF content in *S. virgaurea*, highlighted in our study as a potential source of phenolic compounds, exist. Research with the acetone extract of *S. virgaurea* obtained by traditional extraction was focused on TF and polyphenolic acids contents [22]. Extracts from 10 different locations showed variation in TF levels, with the highest value recorded in the extract from the town of Podkowa Leśna Zachodnia (1380 mg/100 g), due to optimal conditions for growth. Additionally, harvest time significantly influenced TF content; plants harvested in June contained more TF than those harvested in July or September, because the vegetative stage of plant development occurred in that time of year.

For TF content in this study, *T. vulgare* stood out as the richest, and these results are in accordance with findings in the literature. Šukele et al. [23] determined the TP, TF and total phenolic acids content in *T. vulgare* extracts collected from 5 different locations in Latvia. Extracts obtained by maceration were enriched with biologically active compounds, including phenols and flavonoids. The highest TP content was 218.87 ± 4.09 mg GAE/g and the highest TF content was 27.60 ± 0.56 mg QE/g, both from the same location. Furthermore, Ivănescu et al. [24] analyzed the ultrasound-assisted methanol extracts from the aerial parts of *T. vulgare*, *Tanacetum macrophyllum* and *Tanacetum corymbosum*, focusing on TP, TF and total phenolic acids content. *T. vulgare* stood out as the richest in TP (26.37 mg GAE/g DW) and total phenolic acids (0.55 mg CGAE (chlorogenic acid equivalent)/g DW), while *T. macrophyllum* had the lowest levels of all three components. It can be observed that the contents of TP and TF differ in different species of plants within same genus. In the *T. vulgare* extract, TF content was 1.38 mg QE/g DW, significantly lower than the TF content in the ethanol extract from our study (51.85 ± 2.98 mg CE/g DW). The reasons for the significant differences in TF content can be the choice of extraction technique (ultrasounds may cause the degradation of bioactive compounds) as well as the choice of extraction solvent.

#### 2.2.2. Total Content of Hydroxycinnamic Acids (HCA), Flavones (FL) and Condensed Tannins (CT) in Asteraceae Plant’s Extracts

**Figure 3:**
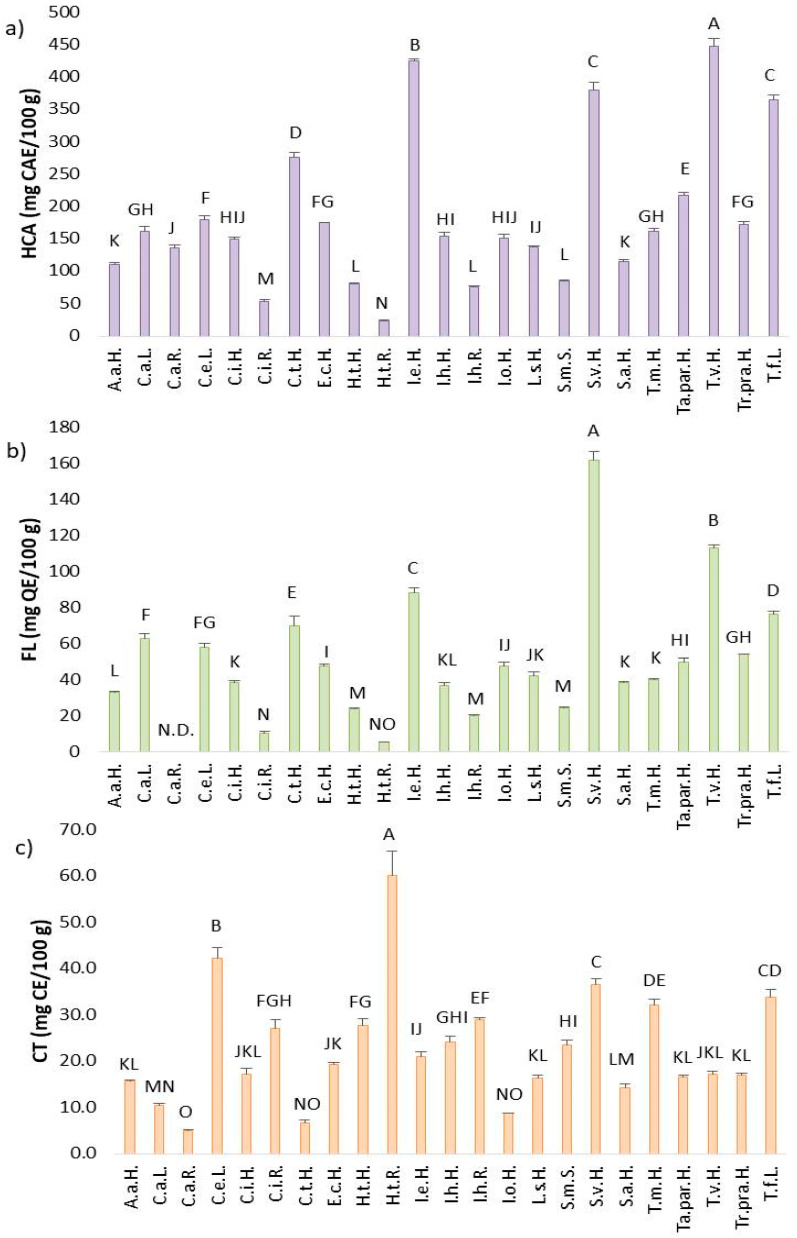
(**a**) Hydroxycinnamic acids (HCA); (**b**) flavones (FL); and (**c**) condensed tannins (CT) contents in Asteraceae plants extracts. Results were expressed as mean ± standard deviation (SD) and different letters represent statistically significant differences (*p* ≤ 0.05) among different plants extracts.

These results are among the first obtained regarding the contents of hydroxycinnamic acids (HCA), flavones (FL) and condensed tannins (CT) across all 23 samples. Hydroxycinnamic acids were the class of bioactive compounds present in all plants, with notably high levels in S.v.H., T.v.H., T.f.L., C.t.H. and I.e.H. (Figure 3a). In contrast, H.t.H., S.m.S., I.h.R., C.i.R., H.t.R., A.a.H., and S.a.H. contained the lowest amounts of HCA. The remaining plants can be considered as having a moderate HCA content. The highest HCA concentration was found in *T. vulgare* (448.12 ± 12.61 mg CAE/100 g), while the lowest was detected in the roots of *H. tuberosus* (23.60 ± 1.10 mg CAE/100 g) (Figure 3a). Hydroxycinnamic acids as a subclass of phenolic acids are mostly synthesized in the aerial parts of the plants, therefore the roots are characterized with lower levels of HCA.

Flavones, which are biologically active compounds classified under flavonoids, ranged from 5.30 ± 0.09 to 161.91 ± 4.72 mg QE/100 g among 23 samples (Figure 3b). *S. virgaurea* had the highest FL content (161.91 ± 4.72 mg QE/100 g), while *H. tuberosus* roots had the lowest. In addition to *S. virgaurea*, high FL levels were also found in *T. vulgare* (112.96 ± 1.82 mg QE/100 g), *Inula ensifolia* (88.38 ± 2.20 mg QE/100 g), *Tussilago farfara* (76.35 ± 1.36 mg QE/100 g), and *Cota tinctoria* (69.57 ± 5.32 mg QE/100 g). Consistent with expectations, the results for five species richest in FL are in correlation with same five species with the highest content of TF, since flavones represent a subclass of flavonoids.

CT represent another important class of biologically active compounds commonly found in Asteraceae plants, including those studied here. Interestingly, although *H. tuberosus* roots were among the poorest in other bioactive compound classes, they showed the highest CT content (60.24 ± 5.16 mg CE/100 g) (Figure 3c). Statistically low CT content was observed in C.a.R., C.t.H., I.o.H., and C.a.L. Moderate CT content was found in T.v.H., I.e.H., E.c.H., C.i.H., Ta.par.H., Tr.pra.H., I.h.H., L.s.H., A.a.H., S.a.H., H.t.H., S.m.S., I.h.R., and C.i.R. Notably high CT content was measured in *C. endivia* (42.31 ± 2.23 mg CE/100 g), *S. virgaurea* (36.68 ± 1.16 mg CE/100 g), *T. farfara* (33.77 ± 1.79 mg CE/100 g), and *T. macrophyllum* (32.25 ± 1.16 mg CE/100 g). Condensed tannins, as a secondary metabolites responsible for plants protection, were primarly present in the aerial parts of Asteraceae plants. The exception was H.t.R. extract with significantly high amount of CT which may be connected to the stage of plant development at which the plant was harvested as well as environmental conditions (stress, light, presence of pathogens) that cause the increased biosynthesis of CT.

It could be concluded that TP represented the class of biologically active compounds with the highest content, while CT was present in the lowest amounts. Among the five less-explored plants (S.v.H., T.v.H., T.f.L., C.t.H., I.e.H.), a consistent trend of high content across various classes of bioactive compounds was observed in comparison to the other samples. Moreover, the TP, TF, FL, and HCA assays all showed aligned results for these five plants. The only exception to this trend was found in the CT assay, where different plants exhibited the highest CT content. In general, the roots of Asteraceae plants contained lower levels of the various bioactive compound classes than their corresponding aerial parts. Given the limited literature on the specific classes of bioactive compounds analyzed in this study, there is a clear opportunity for further research in this area.

### 2.3. In Vitro Antioxidant Activity of Asteraceae Plant’s Extracts

Across the three antioxidant assays conducted, 19 Asteraceae plants investigated in this work showed the highest sensitivity to ABTS radicals, followed by the FRAP assay, and lastly, the DPPH assay.

**Figure 4:**
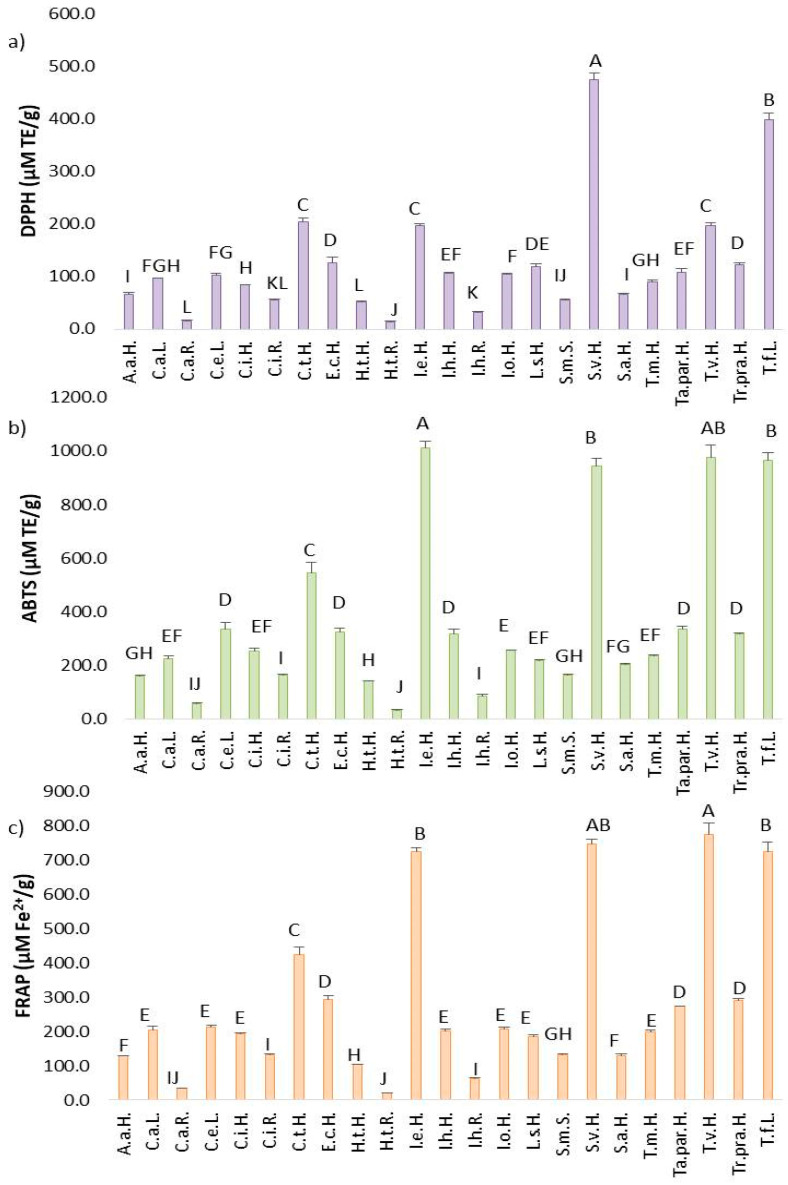
(**a**) DPPH; (**b**) ABTS radical scavenging capacity; and (**c**) ferric reducing antioxidant power (FRAP) of Asteraceae plant extracts. Results are expressed as mean ± standard deviation (SD) and different letters represent statistically significant differences (*p* < 0.05) among different plants extracts.

In the DPPH assay, antioxidant potential among the 23 samples ranged from 14.75 to 475.70 µM TE/g (Figure 4a). Under-researched Asteraceae plants such as *S. virgaurea* (475.70 ± 12.44 µM TE/g), *T. farfara* (399.79 ± 11.64 µM TE/g), *C. tinctoria* (205.33 ± 7.37 µM TE/g), *T. vulgare* (197.81 ± 5.10 µM TE/g)*,* and *I. ensifolia* (197.53 ± 3.33 µM TE/g) demonstrated the highest capacity to neutralize DPPH radicals, due to the highest content of TP and TF as a class of bioactive compounds with pronounced antioxidant activity. The lowest antioxidant potential in this assay was recorded in S.m.S., I.h.R., C.i.R., C.a.R., and H.t.R. The remaining samples exhibited moderate antioxidant activity.

In the ABTS assay, antioxidant activity ranged from 33.02 to 1013.76 µM/g (Figure 4b). The top-performing plants in neutralizing ABTS radicals were *I. ensifolia* (1013.76 ± 22.34 µM TE/g), *T. vulgare* (975.71 ± 45.89 µM TE/g), *T. farfara* (964.76 ± 31.03 µM TE/g), *S. virgaurea* (945.74 ± 27.95 µM TE/g), and *C. tinctoria* (544.22 ± 38.47 µM TE/g), which is associated with the statistically highest TP and TF contents detected in these plants. Conversely, the lowest ability to ABTS-scavenging capacities were measured in C.i.R, I.h.R., C.a.R., and H.t.R., because the root of the species contained a low content of total phenols which are the main agents associated with antioxidant capacity.

Overall, the antioxidant capacity against ABTS radicals was more than twice as high as against DPPH radicals under in vitro conditions. This suggests that different radicals interact with distinct mechanisms of action among the bioactive compounds in the extracts. The majority of antioxidant compounds in the 23 samples appeared to exhibit higher selectivity toward ABTS radicals, due to the chemical composition of extracts in which the bioactive compounds were more selective towards ABTS radicals in comparison to DPPH radicals.

In the FRAP assay, which measures the reduction of Fe^3+^ to Fe^2+^, antioxidant capacity ranged from 19.82 to 774.43 µM Fe^2+^/g by Asteraceae plants (Figure 4c), reflecting intermediate activity levels compared to the DPPH and ABTS assays. Five plants showed strong reducing power: *T. vulgare* (774.44 ± 33.19 µM Fe^2+^/g), *S. virgaurea* (744.39 ± 14.04 µM Fe^2+^/g), *T. farfara* (722.85 ± 27.99 µM Fe^2+^/g), *I. ensifolia* (724.51 ± 8.75 µM Fe^2+^/g), and *C. tinctoria* (424.68 ± 21.13 µM Fe^2+^/g). Twelve plants exhibited moderate values in the FRAP assay, while the lowest values were measured in H.t.H., H.t.R., C.a.R., C.i.R., I.h.R., and S.m.S. Taken together, results from all three antioxidant assays consistently indicated that roots of *H. tuberosus*, *C. acanthifolia utzka*, *C. inthybus*, and *I. helenium* exhibited the weakest antioxidant activity, because the content of TP and TF as the main compounds with antioxidant activity in the root extracts was very low.

There is broad consensus that various plants from the Asteraceae family contain biologically active compounds with pronounced antioxidant activity in their chemical composition [3,25,26,27,28]. For example, the antioxidant potential of extracts from four Asteraceae species—*Onopordum acanthium* L., *Carduus acanthoides* L., *Cirsium arvense* (L.) Scop., and *Centaurea solstitialis* L.—using DPPH radical scavenging assays was evaluated. Three conventional solvents (methanol, ethanol, and acetone) were used for solid–liquid extraction. The highest antioxidant activity was observed in the methanol extract from *C. arvense* leaves, with an IC_50_ value of 366 ng/mL [29]. According to the results it can be concluded that the chemical composition of extracts with antioxidant activity highly depends on the solvent applied in the extraction process.

Among the five plants identified in this study, as having the highest antioxidant potential, *S. virgaurea* has already been recognized as a promising source of antioxidants. One study investigated the antioxidant and antimicrobial activities of *S. virgaurea* extracts obtained using novel extraction techniques, such as accelerated solvent extraction (ASE) and laser irradiation (LE) [30]. The DPPH radical scavenging capacity of *S. virgaurea* extracts (ASE—381.3 ± 2.9 μg/mL, LE—198.4 ± 1.6 μg/mL) exceeded that of the standard compound, vitamin C (39.4 ± 0.1 μg/mL). However, vitamin C showed superior activity in the FRAP assay (125 ± 1.1 μg/mL) in comparison to *S. virgaurea* extracts (ASE—58.67 ± 0.3 μg/mL, LE—56.92 ± 0.4 μg/mL). Similarly, Demir et al. [31] analyzed methanolic and hot water extracts of *S. virgaurea* harvested in Turkey. Antioxidant activity was assessed at various extract concentrations (20–100 µg/mL) using the DPPH assay. The radical scavenging ability increased with concentration, reaching 64.26% in methanol and 30.67% in hot water extracts at 100 µg/mL. The IC_50_ for the methanolic extract was determined to be 74.66 µg/mL. These findings highlight the significant influence of extraction method and solvent type on the biological potential of plant.

*T. vulgare*, another plant with strong antioxidant capacity identified in this study, has also been previously evaluated. Juan-Badaturuge et al. [32] analyzed methanolic extracts of *T. vulgare* aerial parts and reported an IC_50_ value of 37 ± 1.2 µg/mL in the DPPH assay. Furthermore, Ak et al. [33] assessed the antioxidant potential of *T. vulgare* leaves, stems, and aerial parts using maceration with water, ethanol-water mixtures, and hexane. The antioxidant activity varied depending on the solvent, while the non-polar hexane extract exhibited the weakest activity. In contrast, extracts prepared with water or ethanol-water mixtures demonstrated notably strong antioxidant activity in DPPH (124.77 ± 3.16 mg TE/g and 113.92 ± 3.14 mg TE/g), ABTS (172.29 ± 2.08 mg TE/g and 176.94 ± 4.56 mg TE/g), and FRAP (178.57 ± 2.25 mg TE/g and 188.00 ± 0.92 mg TE/g) assays. These studies support the findings of the current research and confirm *T. vulgare* as a plant with promising antioxidant potential for future applications.

*T. farfara* has also been recognized as a rich source of bioactive compounds with notable antioxidant activity, as confirmed by several studies [34,35,36,37]. Bota et al. [34] conducted maceration of *T. farfara* aerial parts collected from two different locations in Romania using 70% ethanol at room temperature for 10 days. The antioxidant capacity, evaluated using CUPRAC, xanthine oxidase inhibition, and FRAP assays, was significantly influenced by geographic origin. Extracts from the Vatra Dornei location showed lower reducing power (55.1 ± 0.5 µM TE/g dry weight) compared to those from the Rarău Mountains (139.7 ± 1.2 µM TE/g dry weight). In another study, Uysal et al. [35] employed a traditional extraction technique to isolate extracts from *T. farfara* L. and *Tragopogon dubius Scop*. using ethyl acetate, methanol, and water. These extracts were tested using ABTS, DPPH, and FRAP assays. Across all three solvents, the ability to scavenge ABTS radicals and the reducing power (FRAP) were consistently higher than the DPPH radical scavenging activity. The ethyl acetate extract exhibited the lowest antioxidant potential in all three assays. Conversely, the methanol extract demonstrated the highest activity, with ABTS (410.98 ± 4.46 mg TE/g extract), DPPH (192.35 ± 0.14 mg TE/g extract), and FRAP (465.31 ± 7.60 mg TE/g extract) values indicating strong antioxidant capacity. An observation can be made that the methanol stood out as the best solvent for isolating extracts from Asteraceae plants with high antioxidant capacity.

Additionally, Dobravalskytė et al. [37] evaluated the antioxidant activity of solid residues of *T. farfara* left after hydrodistillation using acetone, methanol, and ethanol as solvents. Based on IC_50_ values in the DPPH assay, ethanol extracts exhibited the highest antioxidant potential (0.15 ± 0.00 mg/mL), outperforming methanol and acetone extracts. However, the same ethanol extract showed lower activity in the ABTS assay (19.6 ± 0.30%) and the FRAP assay (0.44 ± 0.00 mg/mL) compared to the other solvent extracts. These differences in antioxidant potential are likely due to both climatic variations in the plant collection locations (France and Lithuania) and the choice of extraction solvents.

Very limited scientific data are available regarding the biological activity of *I. ensifolia* extracts. A recent study by Trendafilova et al. [38] investigated six Inula species from Bulgaria, including *I. ensifolia*. Methanol extracts, which were defatted with chloroform prior to analysis, were evaluated for their chemical profile, antioxidant, cytotoxic, and enzyme inhibitory activities. Among the species tested, *I. ensifolia* demonstrated the highest antioxidant activity in the DPPH assay, with a scavenging capacity of 69.41 ± 0.55%. In the ABTS assay, evaluated via the TEAC (Trolox Equivalent Antioxidant Capacity) method, the *I. ensifolia* extract showed an antioxidant capacity of 0.232 ± 0.026 mg/mL. Additionally, another research group from Turkey examined the antioxidant activity of fifteen Inula species [39]. Ethanol extracts were obtained using Soxhlet extraction and assessed for total antioxidant activity. *I. ensifolia* exhibited an antioxidant capacity of 71.27 ± 0.007 µg AAE (ascorbic acid equivalent)/mL.

*C. tinctoria*, commonly known as golden marguerite, is an Asteraceae plant that remains a relatively unexplored source of antioxidant activity. Bahadori et al. [40] investigated the medicinal potential of *C. tinctoria* using both infusions (aerial parts with hot water) and methanol extracts obtained by maceration. Antioxidant capacity was assessed through multiple assays, including total antioxidant capacity, DPPH, ABTS, FRAP, CUPRAC, and metal chelating activity. The methanol extract exhibited the highest total antioxidant capacity (401 mg TE/g extract) compared to the infusion (380 mg TE/g extract). Similarly, the methanol extract showed superior performance in the DPPH (144.94 ± 0.34 mg TE/g extract) and ABTS (275.29 ± 3.16 mg TE/g extract) assays compared to the infusion (DPPH: 125.13 ± 2.66 mg TE/g; ABTS: 192.79 ± 6.82 mg TE/g). However, in the FRAP assay, the infusion demonstrated greater reducing power (292.45 ± 4.96 mg TE/g extract) than the methanol extract (207.43 ± 3.28 mg TE/g extract). Further supporting its antioxidant potential, Meriç et al. [41] evaluated the DPPH radical scavenging activity of *C. tinctoria* methanol extract among 26 extracts from 24 different plant species. The IC_50_ value for *C. tinctoria* (0.038 mg/mL) was found to be comparable to that of standard antioxidants such as ascorbic acid (0.022 mg/mL) and quercetin (0.028 mg/mL), suggesting a high antioxidant capacity. It is important to note that due to taxonomic complexities, golden marguerite may also appear in the literature as *Anthemis tinctoria*. In this context, one study reported that ethyl acetate, methanol, and aqueous extracts of *A. tinctoria* aerial parts obtained by maceration exhibited notable antioxidant activity, further supporting the therapeutic potential of this species [42].

### 2.4. Correlation Between TP, TF, HCA, FL, CT and DPPH, ABTS, FRAP

According to Table 1, the correlation between TP and other subclasses of phenolic compounds such as TF, HCA, FL, and CT were assessed using Pearson’s correlation coefficient (r). Correlations were also analyzed between different phenolic classes and antioxidant potential, measured by DPPH, ABTS, and FRAP assays. A good correlation (*p* < 0.05) was observed between TP and TF, HCA, FL, as well as with antioxidant potential (DPPH, ABTS, FRAP assays), leading to the observation that antioxidant activity is closely related to the high content of a variety of bioactive compounds. In contrast, a negative correlation was found between TP and CT (r = −0.057). A similar trend was observed for TF, HCA, and FL with CT. The main reason for this was the deviation in the H.t.R. sample, which contained an unexpectedly high amount of CT within a low amount of TP. Environmental condition (stress) or the stage of harvesting may be colsely connected with the high CT content in H.t.R., which was not in correlation with other classes of bioactive compounds. Furthermore, CT content showed poor correlation with antioxidant activity (DPPH r = 0.115, ABTS r = 0.028 and FRAP r = −0.000), with no significance (*p* > 0.05). This deviation is likely due to the observation that the plant extract with the highest CT content (rood of *H. tuberosus*) exhibited low antioxidant capacity, while extracts with strong antioxidant activity contained low levels of CT. Among antioxidant assays, DPPH and ABTS assays showed a strong correlation, and the FRAP assay was also highly correlated with DPPH (*p* < 0.05). Notably, a very high correlation (r = 0.994) was found between the FRAP and ABTS assays, indicating a strong relationship between these two methods. Therefore, chemical profiles of the obtained Asteraceae extracts consist of bioactive compounds with targeted activity towards Fe^3+^ reduction and ABTS radicals neutralization.

Similarly, high r values were observed between TF and both ABTS and FRAP assays. This suggests that flavonoids are biologically active compounds contributing significantly to the antioxidant potential of Asteraceae plant extracts. This conclusion aligns with the observed proportion of total flavonoids within the total phenolic content across many samples in this study. Overall, the four classes of bioactive compounds (TP, TF, HCA, FL) demonstrated stronger correlations with ABTS and FRAP assays than with DPPH. Thus, the Asteraceae plant extracts appear to be rich in bioactive compounds with a higher affinity or selectivity for ABTS and FRAP antioxidant assays. A similar affinity for antioxidant assays may be a characteristic of the Asteraceae family that could be utilized for creating various products against oxidative stress.

### 2.5. Chemical Profile of Asteraceae Plant’s Extracts by LC-MS

An extensive screening of the chemical profiles of Asteraceae plants provides insight into the similarities and differences among these plants. Five plant extracts (S.v.H., T.v.H., T.f.L., C.t.H. and I.e.H.) with the highest content of different classes of bioactive compounds were selected for chemical profiling. According to LC-MS analysis, 130 compounds were identified across the Asteraceae family (Table S1). To the best of our knowledge, this represents the most comprehensive identification of bioactive compounds in extracts from these plants to date. The chemical profile included organic acids, hydroxybenzoic acids, hydroxycinnamic acids, flavonoid glycosides, flavonoid aglycones, terpenes and seven compounds classified as other metabolites. Three organic acids (quinic, fumaric and malic acids) were detected in the analyzed samples. Moreover, fifty-five flavonoid glycosides were identified, representing the largest class of bioactive compounds.

A large number of components were present in each sample and may be considered characteristic of the Asteraceae family. The compounds common to all samples (Table S2) included several polar phenolic compounds. Among the hydroxybenzoic acids, the shared compounds were hydroxybenzoic acid hexoside, vanillic acid, dihydroxybenzoic acid hexoside, dihydroxybenzoic acid, dihydroxybenzoic acid ethyl ester, diydroxybenzoic acid caffeoyl-hexoside. Within the hydroxycinnamic acid group, common compounds included 5-*O*-caffeoylquinic acid isomer, 4-*O*-*p*-coumaroylquinic acid, 5-caffeoylshikimic acid, 4-*O*-feruloylquinic acid, disuccinyl-4-*O*-caffeoylquinic acid, 5-*O*-*p*-coumaroylquinic acid, caffeic acid hexoside derivative (Glehnoside), dicaffeoylquinic acid hexoside, dicaffeoylquinic acid isomer 1, dicaffeoylhexose, dicaffeoylhexose, dicaffeoylquinic acid isomer 2, coumaroyl-caffeoylquinic acid, feruloyl-caffeoylquinic acid, tricaffeoylquinic acid and caffeic acid ethyl ester. A variety of phenolic acids derivatives common to all samples may be the main source of the confirmed biological activity of Asteraceae species.

Among flavonoid glycosides, the largest class of compounds, those consistently identified across all samples included: quercetin 3,7-di-*O*-hexoside, myricetin 3-*O*-hexoside, quercetin 3-*O*-(6″rhamnosyl)-hexoside, quercetin 3-*O*-hexoside, kaempferol 3-*O*-hexoside, luteolin 7-*O*-hexuronide, quercetin 3-*O*-(6″-malonyl)-hexoside, quercetin 3-*O*-(6″-acetyl)-hexoside, kaempferol 3-*O*-(6″-malonyl)-hexoside, kaempferol 3-*O*-(6″-acetyl)-hexoside, quercetin 3-*O*-(6″-caffeoyl)-hexoside, and chrysoeriol 7-*O*-hexoside. Therefore, it can be observed that a wide spectrum of flavonoid glycosides was represented by 5 species from Asteraceae family, and the sugar part of the glycosides contains predominantly hexosides. Furthermore, among the flavonoid aglycones, the compounds eriodyctiol, luteolin, quercetin, isorhamnetin, naringenin, and apigenin were consistently detected in all five plants samples. In the group of lipophilic bioactive compounds, the only terpene common to all analyzed samples was herbarumin I. Finally, within the group classified as other metabolites, benzyl pentosyl-hexoside, tuberonic acid hexoside, chrysophanol, hydroxy-octadecadienoic acid, octadecenedioic acid and hydroxyoctadecatri-enoic acid were predominantly identified in *S. virgaurea*, *T. vulgare*, *T. farfara*, *C. tinctoria* and *I. ensifolia*. Even though both five species belong to different genera and have different chemical compositions, there are basically a lot of compounds from different groups of bioactive substances that are common to all samples.

According to the heatmap (Figure S3), samples and bioactive compounds along with the color intensity, which indicates compound abundance, facilitates a more precise identification and screening of chemical profiles. The highest intensity of green, indicating high abundance, was observed in *S. virgaurea* extracts for compounds such as dicaffeoyl-hydroxyquinic acid, quercetin, 4-*O*-feruloylquinic acid, quercetin 3-*O*-(6″-pentosyl)-hexoside, quercetin 3-*O*-pentoside, quercetin 3-*O*-pentoside-7-*O*-rhamnoside, kaempferol 3-*O*-(6″-pentosyl)-hexoside, gibberellin A derivate, 2, 5-caffeoylshikimic acid, 4-*O*-*p*-coumaroylquinic acid, 5-*O*-*p*-coumaroylquinic acid, 5-*O*-caffeoylquinic acid isomer 2, dihydroxybenzoic acid pentosyl-hexoside, solidagenol isomer 2, quercetin 3-*O*-hexoside-7-*O*-rhamnoside, quercetin 3-*O*-rhamnoside, keampferol 3-*O*-rhamnoside, quercetin 3-*O*-(2″-caffeoyl)-pentoside, 4α-formyloxy-18-norgrindelic acid, 2-β-hydroxy-6-depxy-solidagolactone IV-18, 19-olide, solidagenol isomer 1, solidagoic acid H, argophyllin A, gibberellin A derivate 1, viguilenin, gibberellin A derivative 3, and solidagolactone VI. Flavonoid glycosides and solidagolactones (highly specific group of bioactive compounds for the genus *Solidago*), represent very important components in the chemical composition of S.v.H. extracts, which significantly contribute to its strong antioxidant potential.

In *T. vulgare* extracts, compounds with high abundance included hydroxyoctadecatrienoic acid, kaempferol 3-*O*-(6″-caffeoyl)-hexoside, hydroxybenzoic acid, fumaric acid, malic acid, vanillic acid hexoside, vanillic acid, isorhamnetin 3-*O*-hexuronide, chrysoeriol 7-*O*-hexuronide, tuberonic acid hexoside, chrysophanol, luteolin 7-*O*-hexuronide, apigenin 6,8-di-C-hexoside, kaempferol 3-*O*-hexoside-7-*O*-hexuronide, luteolin 7-*O*-(6″-pentosyl)-hexoside, jaceosidin 7-*O*-hexuronide, methyl kaempferol 3-*O*-(6″-acetyl)-hexoside, apigenin 7-*O*-hexuronide, diydroxybenzoic acid caffeoyl-hexoside, isorhamnetin, luteolin, chrysoeriol, apigenin 6-C-hexoside, taxifolin, eupatin, kaempferol 3-*O*-hexoside, jaceosidin, ostadecenedioic acid, caffeoylquinic acid hexoside, naringenin, apigenin, apigenin 7-*O*-(6″-caffeoyl)-hexoside, axillarin, eriodyctiol, and tanetin. The high content of phenolic acids and flavonoids identified in T.v.H. extracts supports the obtained significant antioxidant potential.

The most abundant compounds in *T. farfara* differed and included quinic acid, dicaffeoyl-hydroxyquinic acid, dihydroxybenzoic acid, kaempferol 3-*O*-(6″-rhamnosyl)-hexoside, quercetin, syringic acid, apigenin 6-C-pentoside 8-C-hexoside, gallic acid, methyl 5-*O*-caffeoylquinate, myricetin 3-*O*-pentoside, kaepferol 3-*O*-(2″-coumaroyl)-rhamnoside, 4-*O*-caffeoyl-syringoyl-quinic acid, dicaffeoylquinic acid isomer 3, *p*-coumaric acid, dicaffeoylquinic acid isomer 2, kaempferol 3-*O*-(6″-malonyl)-hexoside, kaempferol 3-*O*-(6″-acetyl)-hexoside, caffeic acid, 6-carboxyl-7-hydroxy-2,3-dimethylchromone, dicaffeoylquinic acid isomer 1, hydroxy-octadecadienoic acid, hydroxyoctadecatri-enoic acid, *p*-coumaric acid hexoside isomer 1, kaempferol, coumaroyl-caffeoylquinic acid, quercetin 3-*O*-hexoside, kaempferol 3-*O*-(6″-caffeoyl)-hexoside, tricaffeoylquinic acid, myricetin 3-*O*-hexoside and octadecenedioic acid. A wide range of dicaffeoylquinic and tricaffeoylquinic acids and flavonoid glycosides distinguishes *T. farfara* as a medically significant plant.

The chemical profile of *I. ensifolia* extracts was predominantly composed of hydroxybenzoic acid hexoside, quercetin 3-*O*-(6″-caffeoyl)-hexoside, quinic acid, dicaffeoyl-hydroxyquinic acid, benzyl pentosyl-hexoside, 4-*O*-caffeoyl-syringoyl-quinic acid, dicaffeoylquinic acid isomer 2, dicaffeoylquinic acid isomer 1, 5-*O*-caffeoylquinic acid isomer 1, hydroxybenyoic acid pentosyl-hexoside, 1-*O*-caffeoylquinic acid, caffeoyl-roseoside, feruloyl-caffeoylquinic acid, luteoil 6-C-hexoside, quercetin 3-*O*-(6″-malonyl)-hexoside, quercetin 3-*O*-(6″-acetyl)-hexoside, hydroxybenzoic acid, 4-*O*-feruloylquinic acid, and caffeic acid hexoside derivative (glehnoside). Caffeoylquinic acid derivatives and flavonoid glycosides stood out as the most abundant compounds in I.e.H. extract which correlated with strong antioxidant activity measured by DPPH, ABTS and FRAP assays. Therefore, there is a great potential for commercialization of *I. ensifolia* extracts as natural agents against oxidative stress.

Finally, chemical profile of *C. tinctoria* included dicaffeoylquinic acid isomer 1, hydroxy-octadecadienoic acid, hydroxyoctadecatri-enoic acid, caffeic acid hexoside, *p*-coumaric acid hexoside isomer 2, succinyl-dicaffeoylquinic acid, sinapoyl-dicaffeoylquinic acid, disuccinyl-dicaffeoylquinic acid, patuletin 7-*O*-hexoside (patulitrin), patuletin 3-*O*-hexuronide, isorhamnetin 3-*O*-(6″-rhamnosyl)-hexoside, 6″-malonyl-patulitrin, patuletin, cirsimaritin, succinyl-tricaffeoylquinic acid, dicaffeoylquinic acid hexoside, patuletin 3-*O*-(6″rhamnosyl)-hexoside, disuccinyl-4-*O*-caffeoulquinic acid, chrysoeriol 7-*O*-hexoside, acacetin, quercetin 3-*O*-hexuronide, dicaffeoylhexose, caffeic acid ethyl estar, 6″-acetyl-patulitrin, herbarumin I, fumaric acid, malic acid, vanillic acid hexoside, vanillic acid, dihydroxybenzoic acid hexoside, quercetin 3-*O*-hexoside-7-*O*-hexuronide, tuberonic acid hexoside, dihydroxybenzoic acid pentoside, dihydroxybenzoic acid ethyl ester, ferulic acid, quercetin 3-*O*-(6″rhamnosyl)-hexoside, axillarin 7-*O*-hexoside (axillaroside), and axillarin 3-*O*-(6″-malonyl)-hexoside. This extensive identification singled out for the first time a large number of phenolic compounds that can change the flow from an unused resource to a highly valuable raw material for future commercialization.

Upon examining the chemical profiles of Asteraceae plant extracts, certain group of bioactive compounds stood out as the most abundant. For example, in *S. virgaurea* extracts, flavonoid glycosides were predominant, encompassing a wide array of compounds. Moreover, hydroxycinnamic acids and terpenes were present in substantial amounts. *T. vulgare* extracts were particularly rich in both flavonoid glycosides and flavonoid aglycones. The high content of flavonoid compounds as well as verified high antioxidant potential may favor *T. vulgare* extracts as natural preservatives. In *T. farfara*, flavonoid glycosides, hydroxybenzoic acids and hydroxycinnamic acids constituted the major components of the chemical profile. *C. tinctoria* extract was characterized by high levels of hydroxycinnamic acids, flavonoid glycosides, organic acids, and hydroxybenzoic acids. Similarly, in *I. ensifolia*, hydroxycinnamic acids, hydroxybenzoic acids and flavonoid glycosides were the most prominent classes of compounds. According to the heatmap analysis, flavonoid glycosides emerged as the most diverse and abundant class of bioactive compounds across all five samples, highlighting the need for more detailed identification within this group. Further quantification of flavonoid glycosides may prioritize the most promising species for utilization as natural sources of bioactive substances for implementation in various products.

Furthermore, an analysis of the five chromatograms and their highest peak areas (Figure S4) revealed a consistent trend in dominant bioactive compounds, supporting the observation of a strong correlation in chemical profiles among the Asteraceae family members. Therefore, members of the Asteraceae family could be considered underutilized sources of natural compounds with medicinal significance.

A specific comparative framework using data from the literature remains challenging due to factors such as plant origin, climate conditions, solvent choice, and extraction technique. However, some similarities in chemical composition of the subsequently mentioned works can be underlined.

Recent study of Fursenco et al. [43] reviewed the phytochemical profile of *S. vigraurea*, summarizing various classes of bioactive compounds. Among C6-C1 glycosides, virgaureoside and leiocarposide were reported. Additionally, a range of phenolic acids, vanillic, gallic, caffeic, chlorogenic, neochlorogenic, 5-*p*-coumaroylquinic, 3,5-, 3,4-, 4,5-di-*O*-caffeoylquinic, ferulic, synapic, 3-hydroxyphenylacetic, 3,4-dihydroxyphenylacetic, homovanilic acids, were identified, which is in agreement with the phenolic acids identified in our work. The study also noted the presence of flavonoids such as rutin, quercetin, kaempferol, eridictyol, naringenin, and hesperetin. Other bioactive classes included triterpene saponins, essential oils (e.g., α- and β-pinene, myrcene, limonene, sabinene), sesquiterpenes (germacrene D, β-caryophyllene, α-humulene), coumarins (umbeliferone (7-hydroxy-coumarin)) and polysaccharides. Hence, the chemical profile of *S. virgaurea* ethanol extract was constructed of 3,5-*O*-dicaffeoylquinic acid, 3,4-*O*-dicaffeoylquinic acid, 3,4,5-*O*-tricaffeoylquinic acid and 4,5-*O*-dicaffeoylquinic acid [44], similarly some isomers of dicaffeoylquinic acids and tricaffeoylquinic acid have been identified in S.v.H.

According to the systematic review of Aćimović and Puvača [45] the chemical profile of *T. vulgare* is highly diverse and varies among its different chemotypes. The essential oil of *T. vulgare* is characterized by terpenes such as 1,8-cineole, α-thujone, camphor, borneol and chrysanthenyl acetate. Additionally, various biologically active metabolites have been identified, including hydroxycinnamic acids (e.g., ferulic acids, chlorogenic acid, neochlorogenic acid, dichlorogenic acid), hydroxycinnamoylquinic acids (caffeoylquinic acids and di-, tri- caffeoylquinic acids), flavonoids (apigenin, hyperoside, kaempferol, luteolin, rutin), flavonoid-*O*-qlucuronides (apigenin-7-*O*-glucuronide, eriodictyol-*O*-glucoronide, luteolin-7-*O*-glucuronide), flavonoid-*O*-glucosides (kaempferol-*O*-(caffeoyl) glucoside, luteolin-7-*O*-glucoside, quercetin-3-*O*-glucoside), and organic acids (citric acid and quinic acid). In general, a high similarity in main bioactive compounds in T.v.H. extract exists, and some differences may originate from different chemotypes of *T. vulgare*.

Using the UHPLC-ESI-MS/MS method, 107 compounds were identified in water, ethanol-water, and hexane extracts of *T. vulgare* [33]. These included 6 hydroxybenzoic acids, 2 hydroxycinnamoyl acids, 14 glycosides and sugar esters, 21 acylquinic acids, 22 flavons, 16 flavonols, 7 flavanons, 11 sesquiterpene lactons, and 3 fatty acids amides. Major phenolic components included caffeic acid, salicylic acid, neochlorogenic and chlorogenic acids, 5-feruloylquinic acid, 3,4-, 3,5- and 4,5- dicaffeoylquinic acids, hesperetin/eriodictyol-*O*-hexuronide, luteolin, quercetin, kaempferol, isorhamnetin, naringenin, and hesperetin. Furthermore, luteolin, luteolin-7-glucoside and chlorogenic acid were identified as the most dominant phenolic compounds in *T. vulgare* flowers by thin-layer chromatography [46]. Luteolin was also among the dominant compounds in extracts from herb of *T. vulgare* obtained in our work.

According to Stoyanova and Perifanova-Nemska [47] leaves of *T. farfara* contain caffeic acid as the most dominant compound, along with other phenolic acids such as 3,4-hydroxybenzoic acid, and ferulic acid. Additionally, the chemical profile includes organic acids such as ascorbic, malic and citric acids. Similarly, caffeic acid, kaempferol, and quercetin were the major components in *T. farfara* methanol extract [48], which correlate with major phenolic compounds in ethanol extracts of *T. farfara* from the Balkan Peninsula. In contrast, LC/MS analysis of four different *T. farfara* extracts identified 21 compounds, with chlorogenic acid and hyperoside being the most abundant. These were followed by caffeic acid, *trans*-*p*-coumaric acid, ellagic acid, salicylic acid, luteolin-7-*O*-glucoside, kaempferol, and carnosol across all samples [34], which implies that the chemical composition varied depending on the part of the plant used for the extraction process. The dominant compounds in T.f.L. extract were slightly different.

Among the predominant phenolic compounds identified in the methanol extract of *T. farfara* were hesperidin, chlorogenic acid, and rosmarinic acid [35]. Furthermore, phenolic compounds such as *trans*-caffeic acid, kaempferol, quercetin, kaempferol-3-*O*-glucoside, loliolide, and *p*-coumaric acid were identified in *T. farfara n*-hexane, ethyl acetate, and methanol extracts using gel filtration [49]. Difference in the main compounds in T.f.L. ethanol extract and the extracts obtained with methanol, ethyl acetate and *n*-hexane indicate that composition of the solvent strongly affects the chemical composition of the extract. In addition, another study reported that ethanol extracts of *T. farfara* contained four caffeoylquinic acids: chlorogenic acid, 3,5-dicaffeoylquinic acid (isochlorogenic acid A), 3,4-dicaffeoylquinic acid (isochlorogenic acid B), and 4,5-dicaffeoylquinic acid (isochlorogenic acid C) [50].

Qualitative LC-MS/MS analysis conducted on eight plants from Turkey revealed that the chemical profile of *C. tinctoria* included 12 compounds, notably three cinnamic acid derivatives, 4-hydroxy benzoic acid, vanillic acid, and several flavonoids such as apigenin, hyperoside, kaempferol, hesperidine, galangin, luteolin, rutin and quercetin [41], which differed from chemical profile in this work primarily due to the location where the plant was collected. A more comprehensive profiling of *A. tinctoria* was previously carried out by Orlando et al. [42] identifying 70 compounds, including various phenolic acid derivatives (caffeoylquinic acids, *p*-coumaroylquinic acids, feruloylquinic acid, di- and tri- caffeoylquinic acid, caffeoylferuloylquinic acids), flavonoid aglycones, flavonoid glycosides, and caffeoyl-*O*-flavonoid conjugates. In golden marguerite (*C. tinctoria*) methanol and water extracts, 23 phenolic compounds were quantified using HPLC-DAD with chlorogenic acid, rutin, apigenin and catechin as the major constituents, followed by hesperidin and quercetin [40], which is not consistent with major compounds identified in ethanol extracts from *C. tinctoria*. This implies that a difference in chemical profile could be related to the selection of solvent for extraction process. Notably, compounds such as vanillin, ferulic acid, *o*-coumaric acid, rosmarinic acid, eriodictyol, and luteolin were absent.

In a comparative study of seven *Inula* species, the content of chlorogenic acid (5-CQA), and four dicaffeoylquinic acid (DCQA) isomers were quantified [38]. Among them, *I. ensifolia* flower methanol extract had the highest content of 5-CQA. Significant levels of 3,4-DCQA, 3,5-DCQA and 4,5-DCQA were also detected, with 1,5-DCQA showing particularly high abundance relative to other species. Furthermore, 15 compounds from the aerial parts of *I. ensifolia* were isolated using traditional extraction (chloroform and methanol), followed by silica gel column chromatography and preparative TLC. These included three quercetin derivatives (isoquercitrin, hyperin, and quercetin-3-O-β-(6″-caffeoylgalactopyranoside)) and four caffeoylquinic acids (chlorogenic acid, and 1,5-, 3,4-, and 3,5-dicaffeoylquinic acids). Quinic acid derivatives, with yields exceeding 1%, were found to be the most abundant components in *I. ensifolia* extract [51].

## 3. Materials and Methods

### 3.1. Plant Material

Nineteen plant species and twenty-tree samples from Asteraceae family were selected for examination (two samples, i.e., leaves or herb and roots, were analyzed for *C. acanthifolia ssp. utzka*, *C. intybus*, *H. tuberosus*, and *I. helenium*). The study encompassed fifteen different plant genera. In some cases—specifically *Tanacetum* and *Inula*, three different species within the same genus were investigated. All plant materials were collected in the territory of Serbia, during the optimal phenophase for each species in 2024, at various locations (coordinates provided in Table S3), and naturally dried in a dark, ventilated area until a constant weight was achieved. Each dried raw material was ground using a blender (Tefal Blendforce, Hong Kong, China), and the mean particle diameter (mm) was measured in accordance with standard pharmacopeia procedures. Moisture content (%) was also determined based on pharmacopeial method, which involves drying at 105 °C until a constant weight is achieved. The main characteristics of all 23 samples, including mean particle diameter and moisture content are also presented in Table S3. All values were within the acceptable limits defined by the pharmacopeia.

### 3.2. Chemicals, Reagents and Standards

All chemicals used in this research are present in Table 2.

Other chemicals, reagents and solvents used in experimental part were of analytical grade.

### 3.3. Experimental Design

The preliminary study included the 23 samples from Asteraceae family, which were subjected to a conventional extraction technique to obtain extracts for further analysis. Different classes of bioactive compounds were determined, including total phenolics (TP), total flavonoids (TF), hydroxycinnamic acids (HCA), flavonols (FL), and condensed tannins (CT). In addition, antioxidant capacity was assessed using DPPH, ABTS, and FRAP assays. The chemical profile of five selected plant extracts was further examined using liquid chromatography–mass spectrometry (LC-MS) for screening of specific bioactive compounds. The experimental design of this preliminary study on under-researched Asteraceae plants is illustrated in Figure S2.

### 3.4. Conventional Solid/Liquid Extraction Technique

For the screening phase of this study, traditional solid/liquid extraction was selected as a simple, low-cost, and widely applicable technique. Choosing environmentally and human-friendly solvent is essential to effectively transfer bioactive compounds from the solid raw material into the liquid phase. Extracts obtained through this conventional method provides valuable insight into the potential of the studied plants. The conventional extraction technique was performed according to the method described earlier [52] with slight modifications. Solid/liquid extraction was carried out under the following conditions: a solid-to-liquid (S/L) ratio of 1:20 (*w*/*v*), using 60% ethanol as the solvent. The extraction was performed with constant shaking on a shaker (IKA KS 4000 I control, IKA, Staufen, Germany) at 180 rpm for 24 h at room temperature. Extracts were prepared in duplicate for each of the 23 different plant materials. Sample labels corresponding to each extract are presented in Table 1. After extraction, the solvent was removed using a rotary vacuum evaporator (IKA RV 05 BASIC 1-B, IKA, Staufen, Germany). The extraction yield was then calculated and expressed as a percentage (%, *w*/*w*).

### 3.5. Classes and Subclasses of Bioactive Compounds

All spectrophotometric methods were performed using a UV/Vis spectrophotometer Shimadzu, model UV-1900i (Shimadzu, Kyoto, Japan), and all results are presented as the mean ± standard deviation of two parallel determinations.

#### 3.5.1. Total Phenolic (TP) and Flavonoid Content (TF)

The determination of total phenolic (TP) and total flavonoid (TF) contents was performed according to the methods described in the literature [53,54]. For TP, results were expressed as mg gallic acid equivalent (GAE) per g of dry weight (mg GAE/g DW). A calibration curve was constructed using gallic acid solutions in the concentration range of 100–700 mg/L (R^2^ = 0.999). For TF, results were expressed as mg of catechin equivalent (CE) per g of dry weight (mg CE/g DW). The calibration curve was prepared using catechin solutions in the concentration range of 25–300 mg/L (R^2^ = 0.9966). Determining the flavonoid content, along with other classes of phenolic compounds, is essential for the future identification and targeting of individual bioactive components.

#### 3.5.2. Total Content of Hydroxycinnamic Acids (HCA), Flavonols (FL) and Condensed Tannins (CT)

For the determination of HCA and FL contents, a slightly modified method from literature was used [55]. A 1:1 mixture (250 µL + 250 µL) of plant extract and HCl solution in 96% ethanol (1 g/L) were mixed, followed by the addition of 4.55 mL of hydrochloric acid dissolved in distilled water (2 g/L). After homogenization using a vortex shaker (IKA vortex genius 3, IKA, Staufen, Germany) for 1 min, the mixture was incubated for 30 min at room temperature in the dark. Absorbance was measured at different wavelengths for HCA (320 nm) and FL (360 nm). For the blank sample, 60% ethanol was used instead of the plant extract and mixed with the reagents following the same procedure. Calibration was performed using chlorogenic acid for HCA (10–600 mg/L, R^2^ = 0.99) and quercetin for FL (10–600 mg/L, R^2^ = 0.9937). Absorbance was measured in duplicate for each sample, and the results were expressed as mg of chlorogenic acid equivalent (CAE) per 100 g of sample (mg CAE/100 g) and mg quercetin equivalent (QE) per 100 g of sample (mg QE/100 g).

The total content of CT was determined using a modified method from the literature [56]. The reaction mixture consisted of 1 mL of plant extract, 2.5 mL of 25% sulfuric acid in methanol, and 2.5 mL of 1% vanillin in methanol. The mixture was homogenized using a vortex shaker (IKA vortex genius 3, IKA, Staufen, Germany) for 1 min. A blank was prepared using 60% ethanol instead of plant extract, following the same procedure. After homogenization, the mixture was incubated at room temperature for 10 min. Absorbance was measured at 500 nm. Calibration was performed using catechin solutions (90–700 mg/L, R^2^ = 0.9989), and the total CT content was expressed as mg catechin equivalent (CA) per 100 g of sample (mg CA/100 g).

### 3.6. In Vitro Antioxidant Activity

In vitro antioxidant activities of 23 samples were evaluated by DPPH, ABTS and FRAP assays.

#### 3.6.1. DPPH Assay

The ability of the 23 samples to scavenge 2,2-diphenyl-1-picrylhydrazyl (DPPH) radicals was assessed using the spectrophotometric method previously described [57]. Calibration was performed using a series of Trolox solutions at concentrations ranging from 6.25 to 200 mg/L (R^2^ = 0.9995). Results were expressed as mg of Trolox equivalent (TE) per g of DW (mg TE/g DW).

#### 3.6.2. ABTS Assay

The antioxidant activity against 2,2′-azino-bis-(-3-ethylbenzothiazoline-6-sulfonic acid) diammonium salt (ABTS) radicals was determined according to the spectrophotometric method [58]. A calibration curve was constructed using Trolox solutions ranging from 6.25 to 200 mg/L (R^2^ = 0.9995). Results were represented as mM of Trolox equivalent (TE) per gram of DW (mM TE/g DW).

#### 3.6.3. FRAP Assay

The ferric reducing antioxidant power (FRAP) assay, which measures the reduction in ferric cation (Fe^3+^) to ferrous cation (Fe^2+^), was carried out based on the method from the literature [59]. A calibration curve was generated using ferrous sulfate heptahydrate solutions in the range of 7.8125 to 500 mg/L (R^2^ = 0.9986). Results were expressed as mg of Fe^2+^ equivalents per g of DW (mg Fe^2+^/g DW).

### 3.7. Chemical Profile of Asteraceae Plant’s Extracts by LC-MS

The chemical analysis of five plant extracts from Asteraceae family (*S. virgaurea*, *T. vulgare*, *T. farfara*, *C. tinctoria*, and *I. ensifolia*) was conducted using LC/MS (Thermo Scientific™ Vanquish™ Core HPLC system) coupled with an Orbitrap Exploris 120 mass spectrometer (San Jose, CA, USA). All LC-MS parameters are previously described in the literature [60]. The obtained MS data were processed and analyzed using RStudio software (version 2023.09.1, build 494) using enviPick and xcms R packages [61]. Identification of bioactive compounds was conducted based on their chromatographic behavior and HRMS/MS^2^ data, with comparisons made to available reference standards and literature data on metabolites from Asteraceae family for tentative identification [62,63,64,65,66,67,68,69,70]. A literature review was performed by searching the SciFinder database [71] using proposed molecular formulas and relevant keywords. Data acquisition was carried out using the Xcalibur^®^ data system (Thermo Finnigan, San Jose, CA, USA).

### 3.8. Statistical Analysis

The results for each assay are expressed as the mean ± standard deviation (SD) of two replicates. To assess significant differences among samples in TP, TF, HCA, FL, and CT contents, as well as in DPPH, ABTS, and FRAP assay results, analysis of variance (ANOVA) followed by Tukey’s HSD post hoc test was applied (*p* < 0.05). Pearson’s correlation coefficient (*r*) was calculated in Minitab 16 (*p* < 0.05) to evaluate the relationships between different classes of phenolic compounds and antioxidant potential. To differentiate between samples, hierarchical cluster analysis (HCA) plots were constructed in Morpheus software (Broad Institute, 2025, https://software.broadinstitute.org/morpheus/, accessed on 10 August 2025) [72], based on the Spearman method of cluster agglomeration, adopting the average linkage method.

## 4. Conclusions

The Asteraceae family, with its vast and underexplored botanical diversity, holds great potential for integration into everyday applications. This preliminary study highlights the selected Asteraceae species as rich natural sources of biologically active compounds, based on their diverse classes of metabolites, antioxidant capacity, and detailed chemical profiles. For the first time, specific plant parts from these species were comprehensively analyzed. Using traditional solid–liquid extraction with ethanol as a green solvent, extraction yields (EY) were determined. However, the results indicate that higher EY does not necessarily correlate with extracts enriched in target compounds.

The contents of various classes of bioactive compounds—total phenols (TP), total flavonoids (TF), hydroxycinnamic acids (HCA), flavonoids (FL), and condensed tannins (CT)—were quantified in 23 samples. Among them, five plant extracts (*Solidago virgaurea*, *Tanacetum vulgare*, *Tussilago farfara*, *Cota tinctoria*, and *Inula ensifolia*) emerged as the most chemically diverse and abundant across all tested parameters. Additionally, their antioxidant potential, evaluated through DPPH, ABTS, and FRAP assays, was found to be positively correlated with the concentration of various bioactive compound classes. The high antioxidant activity observed in these five species supports their potential as natural agents against oxidative stress and as candidates for use as non-synthetic preservatives.

To better understand their phytochemical richness, a detailed LC-MS screening of these five plants was performed, identifying approximately 130 bioactive compounds. While notable differences in composition were observed, all extracts shared a predominance of flavonoid glycosides. Given their established bioactivity, flavonoid glycosides represent a promising group of compounds for targeted isolation from Asteraceae plant extracts.

Although the traditional extraction approach applied here successfully yielded bioactive-rich extracts, the full potential of these plants in terms of application of novel extraction techniques and implementation of improved extracts into different products such as food, cosmetic or pharmaceutical still remains untapped. Future studies should explore advanced green extraction methods, such as Natural Deep Eutectic Solvents (NADES), supercritical fluid extraction, and pressurized liquid extraction, as alternatives to conventional maceration. The current lack of literature on such techniques in this context underscores the need for further research, particularly within the framework of clean-label product development.

Future directions should focus on the targeted quantification of flavonoids and optimization of green extraction methods to enhance their recovery. Extracts rich in flavonoids and proven safe for human use could be incorporated into food, cosmetic, or pharmaceutical products. By bridging natural biodiversity with innovative technologies, these underutilized Asteraceae species offer a promising avenue to address challenges in human health and promote sustainable, functional bioactive ingredients.

## Figures and Tables

**Table 1:** Correlation matrix with Pearson’s coefficient of correlation for TP, TF, HCA, FL, CT, DPPH, FRAP and ABTS.

R	TP	TF	HCA	FL	CT	DPPH	ABTS	FRAP
TF	0.960 0.000							
HCA	0.952 0.000	0.980 0.000						
FL	0.901 0.000	0.882 0.000	0.888 0.000					
CT	−0.057 0.798	−0.020 0.926	−0.115 0.600	−0.094 0.679				
DPPH	0.871 0.000	0.845 0.000	0.807 0.000	0.875 0.000	0.115 0.602			
ABTS	0.968 0.000	0.984 0.000	0.974 0.000	0.874 0.000	0.028 0.897	0.864 0.000		
FRAP	0.971 0.000	0.987 0.000	0.975 0.000	0.894 0.000	−0.000 0.998	0.875 0.000	0.994 0.000	

r—Pearson’s correlation coefficient.

**Table 2:** Chemicals and reagents used in experimental work.

Supplier	Chemicals	Analysis
Sigma-Aldrich Gmbh (Steinheim, Germany)	Folin–Ciocalteu reagent, 1,1-diphenyl-2-picryl-hydrazyl-hydrate (DPPH *), 2,2′-azino-bis-(3-ethylbenzothiazoline-6-sulfonic acid) diammonium salt (ABTS *), 2,4,6-tris (2-pyridyl)-s-triazine (TPTZ, gallic acid, (±)-catechin, (±)-6-hydroxy-2,5,7,8-tetramethylchromane-2-carboxylic acid (Trolox), catechin	TP test TF test DPPH assay ABTS assay FRAP assay CT test
Sani-Hem d.o.o. (Novi Bečej, Serbia)	ethanol 96%	ABTS assay
Acros Organics (Geel, Belgium)	potassium persulfate 99%, quercetin	ABTS assay, FL test
Carlo Erba (Emmendingen, Germany)	methanol 99.5%	DPPH assay
Carl ROTH GmbH + Co (Karlsruhe, Germany)	vanillin 99%	CT assay

* represents radicals.

## Data Availability

The original contributions presented in this study are included in the article/Supplementary Materials. Further inquiries can be directed to the corresponding author.

## Supplementary Materials

The following supporting information can be downloaded at https://www.mdpi.com/article/10.3390/plants14182904/s1: Figure S1. Application, medical properties and therapeutic use of plants from Asteraceae family. Figure S2. Experimental design for under-researched Asteraceae plants. Figure S3. Heatmap of the scaled data of untargeted metabolite analysis, with the samples (both columns and rows) arranged according to the HCA (Spearman method of cluster agglomeration). Intensity of green color indicate the abundance of the compounds in samples. Figure S4. Base peak chromatograms for (a) *S. virgaurea* extract; (b) *T. vulgare* extract; (c) *T. farfara* extract; (d) *C. tinctoria* extract; and (e) *I. ensifolia* extract. Table S1. LC-MS data on metabolites identified in extracts of five plants from Asteraceae family. Table S2. Peak areas of identified compounds, obtained from full scan MS; analyzes were performed in duplicate. Table S3. Under-research plants from Asteraceae family and their main characteristics.
