# Supplementary material for: A Preliminary Insight into Under-Researched Plants from the Asteraceae Family in the Balkan Peninsula: Bioactive Compound Diversity and Antioxidant Potential
Source: Plants (Basel). 2025 Sep 18;14(18):2904. doi: 10.3390/plants14182904 (PMC12473203; doi:10.3390/plants14182904)
Supplement: Supplementary file 1 [file plants-14-02904-s001.zip › plants-3834010-supplementary.pdf]

Supplementary Materials

Table S1. LC-MS data on metabolites identified in extracts of five plants from Asteraceae family.

No	Compound name	t_R, min	Molecular formula, $[M-H]^-$	Calculated mass, *m/z*	Exact mass, *m/z*	Δ mDa	MS² Fragments, (% Base Peak)
	Organic acids						
1	Quinic acid	0.52	$C_7H_{11}O_6^-$	191.05610	191.05698	-0.88	85.02991(23), 87.00916(24), 111.00931(47), 129.02026(4), 191.05698(100)
2	Fumaric acid	0.53	$C_4H_3O_4^-$	115.00370	115.00426	-0.56	71.01415(100), 115.00425(19)
3	Malic acid	0.57	$C_4H_5O_5^-$	133.01420	133.01486	-0.66	71.01414(33), 115.00421(100), 133.01486(50)
	Hydroxybenzoic acids						
4	Dihydroxybenzoic acid pentoside	0.51	$C_{12}H_{13}O_8^-$	285.06159	285.06339	-1.80	108.02209(56), 109.03049(10), 152.01218(100), 153.02013(69), 285.06339(41)
5	Dihydroxybenzoic acid pentosyl-hexoside	0.68	$C_{18}H_{23}O_{13}^-$	447.11442	447.11600	-1.59	108.02202(5), 109.02997(11), 152.01219(100), 153.01981(9)
6	Hydroxybenzoic acid hexoside	0.68	$C_{13}H_{15}O_8^-$	299.07724	299.07989	-2.64	93.03495(34), 137.02502(100)
7	Gallic acid	0.70	$C_7H_5O_5^-$	169.01425	169.01495	-0.70	125.02500(100), 150.97142(24)
8	Vanillic acid hexoside	0.72	$C_{14}H_{17}O_9^-$	329.08781	329.08929	-1.48	108.0222(12), 123.04576(27), 152.01225(17), 167.03575(100)
9	Vanillic acid	0.77	$C_8H_7O_4^-$	167.03498	167.03568	-0.70	108.02241(10), 123.01051(5), 123.04578(100), 152.01216(35), 167.03551(50)
10	Dihydroxybenzoic acid hexoside	0.79	$C_{13}H_{15}O_9^-$	315.07216	315.07350	-1.35	108.02203(13), 109.02998(56), 152.01219(42), 153.02002(100), 315.07367(50)
11	Dihydroxybenzoic acid	0.98	$C_7H_5O_4^-$	153.01933	153.01997	-0.64	109.02999(100), 153.02005(34)
12	Syringic acid	1.20	$C_9H_9O_5^-$	197.04555	197.04634	-0.80	135.04581(100), 152.89417(41), 153.02005(29), 179.03593(22), 182.02284(13), 197.04631(21)

13	Hydroxybenzoic acid	2.10	$C_7H_5O_3^-$	137.02442	137.02501	-0.59	93.03497(7), 137.02501(100)
14	Hydroxybenzoic acid pentosyl-hexoside	4.99	$C_{18}H_{23}O_{12}^-$	431.11950	431.12124	-1.74	71.01411(31), 89.02487(43), 101.02493(35), 125.02504(39), 152.08514(34), 153.09286(100)
15	Dihydroxybenzoic acid ethyl ester	6.35	$C_9H_9O_4^-$	181.05063	181.05135	-0.72	108.02217(23), 109.03004(15), 152.0123(17), 153.02013(39), 181.05151(100)
16	Diydroxybenzoic acid caffeoyl-hexoside	6.44	$C_{22}H_{21}O_{12}^-$	477.10385	477.10580	-1.95	109.02998(12), 152.01216(11), 153.02000(100), 161.02516(33), 179.03584(7), 315.07498(36)
	Hydroxycinnamic acids						
17	1-*O*-Caffeoylquinic acid	1.11	$C_{16}H_{17}O_9^-$	353.08781	353.08885	-1.05	135.04576(12), 173.04596(3), 179.03581(34), 191.05692(100)
18	Caffeic acid hexoside	4.07	$C_{15}H_{17}O_9^-$	341.08781	341.08891	-1.11	135.04578(19), 179.03580(100)
19	5-*O*-Caffeoylquinic acid isomer 1	4.32	$C_{16}H_{17}O_9^-$	353.08781	353.08872	-0.91	191.05690(100)
20	Caffeic acid	4.48	$C_9H_7O_4^-$	179.03498	179.03570	-0.71	135.04578(100), 179.03584(22)
21	*p*-Coumaric acid hexoside isomer 1	4.51	$C_{15}H_{17}O_8^-$	325.09289	325.09429	-1.39	119.05083(50), 163.04083(100)
22	Caffeoylquinic acid hexoside	4.73	$C_{22}H_{27}O_{14}^-$	515.14063	515.14272	-2.09	135.04575(5), 161.02510(8), 173.04628(16), 179.03574(33), 191.05684(100), 353.08716(7)
23	5-*O*-Caffeoylquinic acid isomer 2	5.05	$C_{16}H_{17}O_9^-$	353.08781	353.08893	-1.12	191.05687(100)
24	4-*O*-*p*-Coumaroylquinic acid	5.17	$C_{16}H_{17}O_8^-$	337.09289	337.09440	-1.50	87.00912(6), 93.03494(17), 111.04559(4), 163.04085(12), 173.04626(16), 191.05687(100)
25	5-Caffeoylshikimic acid	5.29	$C_{16}H_{15}O_8^-$	335.07724	335.07877	-1.53	135.04575(49), 137.02498(7), 155.03545(3), 161.02513(24), 173.04648(4), 179.03575(100)
26	*p*-Coumaric acid	5.37	$C_9H_7O_3^-$	163.04007	163.04077	-0.70	119.05075(100), 163.04077(15)
27	4-*O*-Feruloylquinic acid	5.43	$C_{17}H_{19}O_9^-$	367.10346	367.10472	-1.26	93.03494(26), 111.0457(6), 173.04633(17), 191.05687(100), 193.05154(10)

28	4-*O*-Caffeoyl-syringoyl-quinic acid	5.49	$C_{25}H_{25}O_{13}^{-}$	533.13014	533.13214	-2.00	135.0457(90), 155.03552(12), 161.02501(20), 173.04620(100), 179.03571(50), 191.05688(31)
29	Disuccinyl-4-*O*-caffeoylquinic acid	5.50	$C_{24}H_{25}O_{15}^{-}$	553.11997	553.12236	-2.39	117.01985(16), 173.04637(14), 191.05696(100)
30	5-*O*-*p*-Coumaroylquinic acid	5.51	$C_{16}H_{17}O_{8}^{-}$	337.09289	337.09446	-1.57	93.03491(4), 163.04092(4), 173.04659(4), 191.05688(100)
31	*p*-Coumaric acid hexoside isomer 2	5.51	$C_{15}H_{17}O_{8}^{-}$	325.09289	325.09449	-1.60	119.05080(100), 163.04106(16)
32	Dicaffeoyl-hydroxyquinic acid	5.58	$C_{25}H_{23}O_{13}^{-}$	531.11442	531.11650	-2.08	135.04576(43), 161.02516(49), 179.03577(100), 191.05682(8), 339.05225(11)
33	Ferulic acid	5.72	$C_{10}H_{9}O_{4}^{-}$	193.05063	193.05135	-0.72	134.03799(100), 149.06154(90), 178.02823(5), 193.04950(4)
34	Caffeic acid hexoside derivative (Glehnoside)	5.73	$C_{20}H_{25}O_{10}^{-}$	425.14532	425.14665	-1.33	135.04587(4), 161.02512(100), 179.03581(6), 425.14758(20)
35	Dicaffeoylquinic acid hexoside	5.79	$C_{31}H_{33}O_{17}^{-}$	677.17240	677.17463	-2.24	173.04625(28), 179.03578(88), 191.05690(100), 323.07846(38), 335.07867(20), 353.08896(29)
36	Dicaffeoylquinic acid isomer 1	6.11	$C_{25}H_{23}O_{12}^{-}$	515.11950	515.12121	-1.71	135.04576(12), 161.02513(4), 173.04637(11), 179.0358(75), 191.05693(100), 353.08917(11)
37	Succinyl-dicaffeoylquinic acid	6.24	$C_{29}H_{27}O_{15}^{-}$	615.13554	615.13767	-2.13	117.01996(3), 161.02507(3), 173.04636(5), 179.03589(4), 191.05688(100)
38	Dicaffeoylhexose	6.31	$C_{24}H_{23}O_{12}^{-}$	503.11950	503.12206	-2.56	135.04578(34), 161.02515(67), 179.03580(100), 323.07867(7), 341.08923(10)
39	Sinapoyl-dicaffeoylquinic acid	6.28	$C_{36}H_{33}O_{16}^{-}$	721.17748	721.18024	-2.76	161.06152(84), 173.04619(10), 179.03581(61), 191.05695(100), 205.05151(31), 353.08899(41)
40	Dicaffeoylquinic acid isomer 2	6.28	$C_{25}H_{23}O_{12}^{-}$	515.11950	515.12129	-1.79	135.04573(6), 161.02513(5), 173.04633(14), 179.03583(34), 191.05693(100), 353.08948(5)

41	Coumaroyl-caffeoylquinic acid	6.40	$C_{25}H_{23}O_{11}^-$	499.12459	499.12662	-2.04	135.04565(16), 161.0251(16), 163.04077(65), 173.04623(70), 179.03574(67), 191.05692(100)
42	Caffeoyl-roseoside	6.42	$C_{28}H_{35}O_{11}^-$	547.21849	547.22058	-2.10	161.02515(100), 179.03583(17), 387.16782(30)
43	Feruloyl-caffeoylquinic acid	6.51	$C_{26}H_{25}O_{12}^-$	529.13515	529.13727	-2.12	135.04578(6), 161.02501(6), 173.04623(100), 179.03578(36), 191.05695(66), 193.05142(22)
44	Dicaffeoylquinic acid isomer 3	6.57	$C_{25}H_{23}O_{12}^-$	515.11950	515.12148	-1.98	135.0457(15), 161.02502(5), 173.04622(92), 179.03572(100), 191.05687(68), 353.08902(18)
45	Methyl 5-*O*-caffeoylquinate	6.66	$C_{17}H_{19}O_9^-$	367.10346	367.10467	-1.21	99.08195(12), 115.07696(21), 135.04575(32), 143.07196(17), 173.04648(5), 179.03575(100)
46	Disuccinyl-dicaffeoylquinic acid	6.77	$C_{33}H_{31}O_{18}^-$	715.15166	715.15417	-2.51	117.01982(8), 173.04628(7), 191.05688(100)
47	Tricaffeoylquinic acid	6.91	$C_{34}H_{29}O_{15}^-$	677.15127	677.15375	-2.48	161.02513(13), 173.04625(100), 179.03577(82), 191.05693(37), 335.07867(24), 353.08902(67)
48	Succinyl-tricaffeoylquinic acid	6.96	$C_{38}H_{33}O_{18}^-$	777.16721	777.17048	-3.27	135.04572(9), 161.02518(10), 173.04628(100), 179.03578(75), 191.05692(27), 353.08902(91)
49	Caffeic acid ethyl ester	7.02	$C_{11}H_{11}O_4^-$	207.06630	207.06718	-0.88	134.03787(16), 135.04572(17), 161.02518(15), 179.03581(29), 207.06717(100)
	Flavonoid glycosides						
50	Quercetin 3-*O*-hexoside-7-*O*-hexuronide	4.79	$C_{27}H_{27}O_{18}^-$	639.12036	639.12312	-2.76	151.00427(4), 178.99974(4), 300.02881(66), 301.03653(55), 463.09018(100)
51	Apigenin 6,8-di-*C*-hexoside	4.92	$C_{27}H_{29}O_{15}^-$	593.15119	593.15337	-2.17	353.06757(100), 383.07874(68), 413.09247(11), 473.11072(47), 503.12161(14), 593.15186(28)

52	Quercetin 3-*O*-hexoside-7-*O*-rhamnoside	5.18	$C_{27}H_{29}O_{16}^{-}$	609.14618	609.14852	-2.34	300.02774(17), 301.03662(100), 446.09027(5), 447.09659(19), 462.08218(87), 463.09085(24)
53	Quercetin 3-*O*-pentoside-7-*O*-rhamnoside	5.31	$C_{26}H_{27}O_{15}^{-}$	579.13554	579.13819	-2.64	300.02841(13), 301.03668(93), 432.07178(100), 433.07962(42), 447.09534(10)
54	Quercetin 3,7-di-*O*-hexoside	5.47	$C_{27}H_{29}O_{17}^{-}$	625.14110	625.14391	-2.81	151.00401(3), 178.99947(3), 300.02884(78), 301.03650(100), 463.09012(34)
55	Apigenin 6-*C*-pentoside 8-*C*-hexoside	5.47	$C_{26}H_{27}O_{14}^{-}$	563.14063	563.14280	-2.17	353.06775(100), 383.07883(89), 413.08978(16), 443.10049(53), 473.10986(38), 503.12228(13)
56	Myricetin 3-*O*-hexoside	5.51	$C_{21}H_{19}O_{13}^{-}$	479.08311	479.08518	-2.07	316.02365(100), 317.0311(13)
57	Luteolin 6-*C*-hexoside	5.53	$C_{21}H_{19}O_{11}^{-}$	447.09329	447.09520	-1.91	285.04205(8), 297.04178(11), 299.05823(7), 327.05252(100), 339.05063(7), 357.06323(44)
58	Quercetin 3-*O*-(6''-pentosyl)-hexoside	5.67	$C_{26}H_{27}O_{16}^{-}$	595.13053	595.13269	-2.16	151.00414(4), 178.99939(4), 300.02887(81), 301.03378(100)
59	Kaempferol 3-*O*-hexoside-7-*O*-hexuronide	5.69	$C_{27}H_{27}O_{17}^{-}$	623.12545	623.12877	-3.33	285.04156(100), 447.09512(8), 461.07364(6)
60	Myricetin 3-*O*-pentoside	5.74	$C_{20}H_{17}O_{12}^{-}$	449.07255	449.07419	-1.64	178.99973(3), 271.02637(3), 316.02380(100), 317.03137(16)
61	Quercetin 3-*O*-(6''rhamnosyl)-hexoside	5.76	$C_{27}H_{29}O_{16}^{-}$	609.14618	609.14845	-2.27	178.99947(3), 300.02869(100), 301.03625(29), 609.14844(10)
62	Apigenin 6-*C*-hexoside	5.77	$C_{21}H_{19}O_{10}^{-}$	431.09837	431.09985	-1.48	283.06250(17), 311.05756(100), 341.06805(9)
63	Patuletin 3-*O*-(6''rhamnosyl)-hexoside	5.77	$C_{28}H_{31}O_{17}^{-}$	639.15675	639.15895	-2.20	315.01605(21), 316.02380(15), 330.03949(100), 331.04715(37), 639.15924(13)
64	Luteolin 7-*O*-(6''-pentosyl)-hexoside	5.78	$C_{26}H_{27}O_{15}^{-}$	579.13554	579.13804	-2.50	285.04169(100), 579.13812(15)
65	Quercetin 3-*O*-hexoside	5.86	$C_{21}H_{19}O_{12}^{-}$	463.08820	463.08990	-1.70	151.00438(84), 300.02893(100), 301.03647(40)

66	Quercetin 3-*O*-hexuronide	5.88	$C_{21}H_{17}O_{13}^{-}$	477.06746	477.06975	-2.29	151.00444(8), 161.02519(11), 178.99948(8), 301.03665(100)
67	Kaempferol 3-*O*-hexoside	5.92	$C_{21}H_{19}O_{11}^{-}$	447.09329	447.09489	-1.60	255.03111(11), 284.03378(100), 285.04150(29)
68	Patuletin 7-*O*-hexoside (Patulitrin)	5.92	$C_{22}H_{21}O_{13}^{-}$	493.09877	493.10087	-2.10	169.01509(12), 315.01608(32), 316.02386(24), 330.03955(100), 331.04700(39)
69	Patuletin 3-*O*-hexuronide	5.92	$C_{22}H_{19}O_{14}^{-}$	507.07803	507.07992	-1.90	301.03354(7), 302.03491(10), 316.0239(91), 331.04749(100)
70	Kaempferol 3-*O*-(6''-rhamnosyl)-hexoside	5.92	$C_{27}H_{29}O_{15}^{-}$	593.15119	593.15320	-2.01	151.0045(3), 255.03099(3), 257.04675(3), 284.03372(72), 285.04141(100), 593.15338(13)
71	Luteolin 7-*O*-hexuronide	5.95	$C_{21}H_{17}O_{12}^{-}$	461.07255	461.07430	-1.75	285.04150(100)
72	Quercetin 3-*O*-(6''-malonyl)-hexoside	6.00	$C_{24}H_{21}O_{15}^{-}$	549.08859	549.09080	-2.20	151.00449(3), 178.9994(3), 271.02542(3), 300.02869(100), 301.03635(37), 505.10074(4)
73	Quercetin 3-*O*-(6''-acetyl)-hexoside	6.01	$C_{23}H_{21}O_{13}^{-}$	505.09877	505.10063	-1.86	151.00397(3), 178.99969(3), 271.02606(3), 300.02881(100), 301.03644(24)
74	Kaempferol 3-*O*-(6''-pentosyl)-hexoside	6.02	$C_{26}H_{27}O_{15}^{-}$	579.13554	579.13782	-2.28	151.00429(2), 284.03384(53), 285.04156(100), 447.09589(5), 579.13849(12)
75	Quercetin 3-*O*-pentoside	6.03	$C_{20}H_{17}O_{11}^{-}$	433.07764	433.07915	-1.51	300.02884(100), 301.03656(39)
76	Isorhamnetin 3-*O*-(6''-rhamnosyl)-hexoside	6.04	$C_{28}H_{31}O_{16}^{-}$	623.16183	623.16426	-2.42	299.02094(16), 300.02884(26), 314.04465(69), 315.05225(100)
77	Quercetin 3-*O*-rhamnoside	6.16	$C_{21}H_{19}O_{11}^{-}$	447.09329	447.09510	-1.81	151.00447(4), 178.99931(4), 300.02881(100), 301.03650(67), 447.09491(4)
78	Isorhamnetin 3-*O*-hexuronide	6.17	$C_{22}H_{19}O_{13}^{-}$	491.08311	491.08483	-1.71	113.02489(8), 300.02869(66), 315.05215(100)
79	6''-Malonyl-patulitrin	6.20	$C_{25}H_{23}O_{16}^{-}$	579.09916	579.10123	-2.07	315.01581(19), 316.02383(24), 330.03952(44), 331.04733(100), 535.11139(8)

80	6''-Acetyl-patulitrin	6.21	$C_{24}H_{23}O_{14}$–	535.10933	535.11060	-1.27	165.99089(3), 181.01498(16), 315.01596(33), 316.02377(33), 330.03955(59), 331.04730(100)
81	Apigenin 7-*O*-hexuronide	6.26	$C_{21}H_{17}O_{11}$–	445.07764	445.07924	-1.61	85.02985(13), 113.02493(16), 201.04137(21), 269.04666(100)
82	Kaempferol 3-*O*-(6''-malonyl)-hexoside	6.30	$C_{24}H_{21}O_{14}$–	533.09368	533.09568	-2.00	255.03073(3), 284.03357(100), 285.04138(83)
83	Kaempferol 3-*O*-(6''-acetyl)-hexoside	6.30	$C_{23}H_{21}O_{12}$–	489.10385	489.10576	-1.91	255.03096(5), 284.03384(100), 285.0416(74)
84	Axillarin 7-*O*-hexoside (Axillaroside)	6.34	$C_{23}H_{23}O_{13}$–	507.11442	507.11617	-1.75	165.99156(5), 181.01511(8), 301.03650(11), 316.02383(31), 329.03183(100), 344.05502(94)
85	Quercetin 3-*O*-(6''-caffeoyl)-hexoside	6.36	$C_{30}H_{25}O_{15}$–	625.11989	625.12207	-2.18	151.00438(4), 161.02507(7), 178.99931(5), 300.02887(100), 301.03653(87), 463.09027(61)
86	Chrysoeriol 7-*O*-hexuronide	6.40	$C_{22}H_{19}O_{12}$–	475.08820	475.09004	-1.84	113.02489(18), 284.03381(18), 299.05728(100)
87	Chrysoeriol 7-*O*-hexoside	6.40	$C_{22}H_{21}O_{11}$–	461.10894	461.11061	-1.68	113.02493(25), 285.04169(100), 446.08716(12)
88	Kaempferol 3-*O*-rhamnoside	6.44	$C_{21}H_{19}O_{10}$–	431.09837	431.09975	-1.37	255.03116(4), 284.03387(33), 285.03830(100)
89	Kaempferol 3-*O*-(6''-caffeoyl)-hexoside	6.56	$C_{30}H_{25}O_{14}$–	609.12505	609.12738	-2.33	161.02516(21), 179.03589(15), 284.03400(34), 285.04166(100), 323.07892(17), 447.09546(8)
90	Jaceosidin 7-*O*-hexuronide	6.59	$C_{23}H_{21}O_{13}$–	505.09877	505.10108	-2.32	113.02488(29), 151.00432(28), 287.05667(17), 314.04462(79), 329.06818(100)
91	Axillarin 3-*O*-(6''-malonyl)-hexoside	6.60	$C_{26}H_{25}O_{16}$–	593.11488	593.11744	-2.56	161.02530(8), 179.03616(3), 287.02106(14), 315.01541(7), 330.03952(84), 345.06274(100)
92	Apigenin 7-*O*-(6''-caffeoyl)-hexoside	6.75	$C_{30}H_{25}O_{13}$–	593.13014	593.13241	-2.27	135.04578(4), 161.02509(33), 179.03584(13), 269.04660(100), 323.07861(23)

93	Quercetin 3-*O*-(2''-caffeoyl)-pentoside	6.88	$C_{29}H_{23}O_{14}^-$	595.10933	595.11115	-1.82	135.04590(6), 161.02493(8), 178.99925(7), 179.03568(20), 300.02872(86), 301.03647(100)
94	Methyl kaempferol 3-*O*-(6''-acetyl)-hexoside	7.29	$C_{24}H_{23}O_{12}^-$	503.11959	503.12219	-2.60	173.04655(26), 284.03369(22), 285.04178(100), 399.07330(16), 443.06506(6), 488.09741(11)
95	Kaempferol 3-*O*-(2''-coumaroyl)-rhamnoside	7.63	$C_{30}H_{25}O_{12}^-$	577.13512	577.13757	-2.45	119.05068(3), 145.03011(10), 163.04077(7), 284.03375(59), 285.04150(100)
	Flavonoid aglycones						
96	Taxifolin	5.80	$C_{15}H_{11}O_7^-$	303.05103	303.05235	-1.33	125.02497(100), 151.04077(8), 175.04092(19), 177.02016(15), 217.05153(15), 285.04190(40)
97	Eriodyctiol	6.77	$C_{15}H_{11}O_6^-$	287.05611	287.05739	-1.27	107.01424(7), 135.04570(68), 151.00429(100)
98	Luteolin	6.87	$C_{15}H_9O_6^-$	285.04046	285.04176	-1.30	151.00424(3), 285.04163(100)
99	Patuletin	6.88	$C_{16}H_{11}O_8^-$	331.04594	331.04752	-1.58	165.99156(19), 166.99976(3), 181.01486(8), 271.02676(6), 287.02109(12), 316.02393(100)
100	Quercetin	6.88	$C_{15}H_9O_7^-$	301.03538	301.03675	-1.38	107.01428(4), 121.03008(13), 151.00432(100), 178.99934(45), 301.03659(74)
101	Isorhamnetin	6.92	$C_{16}H_{11}O_7^-$	315.05103	315.05242	-1.40	255.03163(3), 271.02570(5), 300.02866(100)
102	Axillarin	7.07	$C_{17}H_{13}O_8^-$	345.06159	345.06277	-1.18	287.02100(5), 315.01587(73), 330.03931(100)
103	Naringenin	7.24	$C_{15}H_{11}O_5^-$	271.06120	271.06215	-0.95	107.01427(10), 119.05076(35), 151.00435(100), 165.01962(3), 177.02016(12)
104	Apigenin	7.27	$C_{15}H_9O_5^-$	269.04555	269.04673	-1.19	151.00438(3), 225.05649(3), 269.04663(100)
105	Kaempferol	7.33	C15H9O6−	285.04046	285.04165	-1.19	151.00432(6), 257.04669(5), 285.04160(100)
106	Chrysoeriol	7.36	$C_{16}H_{11}O_6^-$	299.05611	299.05737	-1.26	284.03369(100), 299.05746(9)

107	Jaceosidin	7.41	$C_{17}H_{13}O_7^-$	329.06668	329.06811	-1.43	271.02609(11), 285.04163(7), 286.04932(4), 299.02100(91), 314.04465(100), 329.06830(19)
108	Eupatin	7.62	$C_{18}H_{15}O_8^-$	359.07724	359.07851	-1.27	301.03674(26), 329.03174(100), 344.05502(88)
109	Tanetin	7.95	$C_{18}H_{15}O_7^-$	343.08233	343.08356	-1.23	313.03671(14), 314.04019(88), 328.05902(13), 329.06369(100)
110	Acacetin	8.27	$C_{16}H_{11}O_5^-$	283.06120	283.06245	-1.26	268.03903(100), 283.06274(88)
111	Cirsimaritin	8.55	$C_{17}H_{13}O_6^-$	313.07176	313.07302	-1.26	255.03091(30), 269.04703(4), 283.02600(67), 298.04950(100), 313.07321(25)
	Terpenes						
112	Argophyllin A	7.52	$C_{20}H_{27}O_7^-$	379.17623	379.17749	-1.27	72.99342(57), 273.18723(24), 289.18225(22), 317.17725(60), 335.18848(16), 379.17770(100)
113	4α-Formyloxy-18-norgrindelic acid	7.55	$C_{20}H_{29}O_5^-$	349.20200	349.20326	-1.25	83.05053(33), 137.09773(40), 245.19212(17), 257.19211(100), 275.20261(91), 305.21347(7)
114	Herbarumin I	7.58	$C_{12}H_{19}O_4^-$	227.12888	227.12993	-1.05	165.12926(12), 183.13986(100)
115	Gibberellin A derivative 1	7.92	$C_{20}H_{25}O_6^-$	361.16566	361.16692	-1.26	273.18726(15), 317.17709(100), 361.16714(13)
116	Gibberellin A derivative 2	8.01	$C_{20}H_{27}O_6^-$	363.18137	363.18263	-1.26	255.17641(83), 273.1871(79), 283.17148(23), 301.18210(42), 319.19250(25), 363.18265(100)
117	2-β-Hydroxy-6-deoxy-solidagolactone IV-18, 19-olide	8.24	$C_{20}H_{25}O_5^-$	345.17075	345.17177	-1.03	255.17683(16), 257.19244(45), 273.18848(3), 301.18234(100), 317.17679(4), 345.17242(6)
118	Viguilenin	8.35	$C_{20}H_{29}O_7^-$	381.19188	381.19320	-1.32	275.20303(22), 301.18207(99), 321.17184(25), 337.20319(27), 345.17191(70), 381.19333(100)
119	Solidagenol isomer 1	8.37	$C_{20}H_{29}O_4^-$	333.20713	333.20861	-1.47	83.05058(13), 259.20776(23), 271.20779(92), 289.21851(100), 305.21359(6), 333.20874(39)

120	Gibberellin A derivative 3	8.80	$C_{20}H_{27}O_6$–	363.18131	363.18248	-1.17	257.19226(19), 275.20282(24), 301.18207(100), 319.19257(4), 345.17200(29), 363.18225(3)
121	Solidagenol isomer 2	9.10	$C_{20}H_{29}O_4$–	333.20713	333.20864	-1.50	257.19235(11), 259.20786(100), 289.21841(23), 333.20868(37)
122	Solidagoic acid H	9.25	$C_{20}H_{27}O_5$–	347.18640	347.18735	-0.96	257.19229(16), 259.17197(11), 259.20724(100), 285.18713(37), 303.19781(26), 347.18765(23)
123	Solidagolactone VI	10.16	$C_{22}H_{31}O_5$–	375.21770	375.21894	-1.24	241.19720(71), 257.19299(10), 285.18732(100), 302.19034(13), 331.22986(12), 375.21841(11)
	Other metabolites						
124	Benzyl pentosyl-hexoside*	5.00	C19H27O_{12}–	447.15080	447.15245	-1.65	71.01416(45), 101.02483(73), 113.02494(30), 161.04634(87), 269.10434(100), 401.14703(33)
125	Tuberonic acid hexoside	5.16	$C_{18}H_{27}O_9$–	387.16606	387.16743	-1.37	59.01404(100), 89.02477(33), 101.02484(21), 207.10350(32), 387.16760(46)
126	6-Carboxyl-7-hydroxy-2,3-dimethylchromone	7.71	$C_{12}H_9O_5$–	233.04550	233.04660	-1.10	189.05650(100), 233.04660(31)
127	Chrysophanol	8.24	$C_{15}H_9O_4$–	253.05063	253.05175	-1.11	253.05157(100)
128	Hydroxy-octadecadienoic acid	9.87	$C_{18}H_{31}O_3$–	295.22787	295.22901	-1.14	171.10349(49), 183.10364(4), 195.13992(9), 277.21854(100), 295.22876(72)
129	Octadecenedioic acid	10.01	$C_{18}H_{31}O_4$–	311.22278	311.22404	-1.26	293.21240(3), 311.16995(100)
130	Hydroxy-octadecatrienoic acid	10.02	$C_{18}H_{29}O_3$–	293.21222	293.21343	-1.21	113.09771(13), 249.22357(4), 293.21347(100)

*HCOOH adduct.

Table S2. Peak areas of identified compounds, obtained from full scan MS; analyzes were performed in duplicate.

No	Compound name	*Solidago virgaurea_1*	*Solidago virgaurea_2*	*Tanacetum vulgare_1*	*Tanacetum vulgare_2*	*Tussilago farfara_1*	*Tussilago farfara_2*	*Cota tinctoria_1*	*Cota tinctoria_2*	*Inula ensifolia_1*	*Inula ensifolia_2*
Organic acids											
**1**	**Quinic acid**	6909539497	7531066355	3258881187	3602964066	9740226010	8208024773	5600884945	7069172416	8771356245	6805083392
**2**	**Fumaric acid**	561400729	537895239	681533360	738513840	569949464	678878793	526968199	802408765	604981023	432422361
**3**	**Malic acid**	2785386185	2635133326	3311439110	3622154901	2641904933	2690937164	2473781235	4042227561	3005859230	2047000495
Hydroxybenzoic acids											
**4**	**Dihydroxybenzoic acid pentoside**	9417439	9385975	328030	5116364	0	0	148547444	315053508	7580527	5694128
**5**	**Dihydroxybenzoic acid pentosyl-hexoside**	22293252	26738364	0	0	6846512	8575482	0	0	0	0
**6**	**Hydroxybenzoic acid hexoside**	12162845	16211809	11860054	10156514	24403617	13131743	10505743	3307136	65324602	83964732
**7**	**Gallic acid**	0	0	0	0	60542476	54196178	0	0	0	0
**8**	**Vanillic acid hexoside**	37384414	42272421	57572119	60107967	0	0	61646642	73133660	31254776	35299317
**9**	**Vanillic acid**	9460842	12482268	20484334	21767690	5159452	11088399	22160736	23894753	10999361	17909591
**10**	**Dihydroxybenzoic acid hexoside**	214319655	233182315	486365719	584557473	153532617	262586505	1236803947	1485791345	261559251	224324019
**11**	**Dihydroxybenzoic acid**	84178726	127045183	51579078	79829069	330536980	333676846	271453505	220742114	14216043	40456118

**12**	**Syringic acid**	0	0	0	0	24767190	27980617	0	0	0	0
**13**	**Hydroxybenzoic acid**	52013883	64414961	57323605	98717612	0	0	0	0	86067783	91773738
**14**	**Hydroxybenzoic acid pentosyl-hexoside**	9163824	10612390	0	0	0	0	158833138	206028559	541974041	322143474
**15**	**Dihydroxybenzoic acid ethyl ester**	67136204	87314491	50555662	60530287	14138136	26018179	251890384	242043107	62320230	42289638
**16**	**Diydroxybenzoic acid caffeoyl-hexoside**	26137062	23248971	2891461830	3220484542	99056100	1084317103	701891843	884801069	1355592616	983936936
Hydroxycinnamic acids											
**17**	**1-*O*-Caffeoylquinic acid**	15415491	13539161	0	0	0	0	4782836135	6098784496	9201870102	3591703483
**18**	**Caffeic acid hexoside**	40763973	43372930	48081381	53484587	0	0	269746910	379961310	126503249	69899658
**19**	**5-*O*-Caffeoylquinic acid isomer 1**	11627998641	10711314499	12442470581	13792349762	14399540302	17089363687	13080165268	17944145522	20356465507	14692689478
**20**	**Caffeic acid**	0	0	37211403	43295473	324164072	443157472	240292306	275005631	0	0
**21**	***p*-Coumaric acid hexoside isomer 1**	5444085	6365375	9276690	15086416	22253389	49347808	0	0	0	0
**22**	**Caffeoylquinic acid hexoside**	0	0	47685122	54978530	16641646	26705331	22397369	47285459	0	0
**23**	**5-*O*-Caffeoylquinic acid isomer 2**	2681467921	2504133038	0	0	1039725843	1982227841	0	0	0	0

**24**	**4-*O*-*p*-Coumaroylquinic acid**	1754749789	1758026491	128202124	153602483	454730254	350860865	46535660	61046868	183152192	119974633
**25**	**5-Caffeoylshikimic acid**	591816938	536169633	25193221	29660492	52428850	43642606	23409502	31208810	29354741	22103372
**26**	***p*-Coumaric acid**	0	0	0	0	363559755	263904166	0	0	13029404	11536669
**27**	**4-*O*-Feruloylquinic acid**	952236023	937455522	730734901	819984740	166734786	334612473	439374287	718256866	1263462568	778871003
**28**	**4-*O*-Caffeoyl-syringoyl-quinic acid**	0	0	211098684	240394640	673619156	586067137	143988840	49890699	583056034	367451628
**29**	**Disuccinyl-4-*O*-caffeoylquinic acid**	4251445	2858081	35065985	39160110	24113138	37511754	424929227	736554242	209474669	74834479
**30**	**5-*O*-*p*-Coumaroylquinic acid**	1021323998	991123462	101429328	120591059	346487568	292060562	38844758	55754812	73014395	48984632
**31**	***p*-Coumaric acid hexoside isomer 2**	0	0	0	0	0	0	66000611	102366231	13638730	1889975
**32**	**Dicaffeoyl-hydroxyquinic acid**	228754080	223106924	0	0	199993198	197388161	89162310	165824413	233006771	23230353
**33**	**Ferulic acid**	10001925	11056924	0	0	2272891	4478401	64375352	65652327	0	0
**34**	**Caffeic acid hexoside derivative (Glehnoside)**	11199696	10734816	86745910	121882124	4773738	23462885	78053489	73069601	954638172	831231647

**35**	**Dicaffeoyl quinic acid hexoside**	17343090	11052707	224806481	255218713	106674368	152288674	402496949	801958630	326140056	158422839
**36**	**Dicaffeoyl quinic acid isomer 1**	937663921	916822321	8794319807	9517914114	16352540148	14678258526	12562871363	14859621246	14026949824	11854590945
**37**	**Succinyl-dicaffeoyl quinic acid**	0	0	0	0	0	0	5515224209	7565860862	892869741	110416138
**38**	**Dicaffeoyl hexose**	20850233	23525217	5177990	5219743	27947456	29913500	365472886	438741146	72268797	27475140
**39**	**Sinapoyl-dicaffeoyl quinic acid**	0	0	0	0	0	0	536864474	755156111	312061565	31555183
**40**	**Dicaffeoyl quinic acid isomer 2**	2105702389	2157831929	4636387909	4737143334	11674316580	10055699638	4857822476	5698560951	9347441606	7494465780
**41**	**Coumaroyl-caffeoylquinic acid**	93627530	98710915	81722325	100197584	761278109	589833569	53504675	58683076	165533451	126748679
**42**	**Caffeoyl-roseoside**	0	0	11568414	16351756	0	0	168061858	165213100	488852269	437539270
**43**	**Feruloyl-caffeoylquinic acid**	35334400	33574209	125805416	151566088	56677984	79611500	291783739	358105772	853744730	638362758
**44**	**Dicaffeoyl quinic acid isomer 3**	0	0	1499756783	1737577996	2073373758	2589298074	1114847117	988041651	1775662878	1813098466
**45**	**Methyl 5-*O*-caffeoylquinate**	0	0	0	0	111266788	108491491	0	0	0	0
**46**	**Disuccinyl-dicaffeoyl quinic acid**	0	0	0	0	0	0	371559081	549067221	60368176	7081903
**47**	**Tricaffeoyl quinic acid**	14681504	10977625	219404392	245928986	1399942605	1004210869	144775943	146834775	174221748	114164137

**48**	**Succinyl-tricaffeoyl quinic acid**	0	0	7203186	4332851	1446644	3064329	489875929	758190886	94870368	6488240
**49**	**Caffeic acid ethyl ester**	85060443	72764356	217571933	191260881	914711658	599776074	1424901110	1413755558	1029757382	937123929
Flavonoid glycosides											
**50**	**Quercetin 3-*O*-hexoside-7-*O*-hexuronide**	0	0	19055664	23013250	0	0	22064557	51767706	0	0
**51**	**Apigenin 6,8-di-*C*-hexoside**	0	0	32209185	43651213	0	0	0	0	0	0
**52**	**Quercetin 3-*O*-hexoside-7-*O*-rhamnoside**	40029756	37463313	0	0	25148205	15711513	0	0	0	0
**53**	**Quercetin 3-*O*-pentoside-7-*O*-rhamnoside**	16388162	18254418	0	0	0	0	2769747	3144911	0	0
**54**	**Quercetin 3,7-di-*O*-hexoside**	22564667	13626023	116509064	132767859	21983756	43903068	112947204	233756889	143762864	75644103
**55**	**Apigenin 6-*C*-pentoside 8-*C*-hexoside**	0	0	0	0	11685784	22382853	0	0	0	0
**56**	**Myricetin 3-*O*-hexoside**	5167627	5410012	86639193	76450546	204265954	159671249	42105292	63859525	6630005	8766168
**57**	**Luteolin 6-*C*-hexoside**	0	0	0	0	0	0	45605057	40461014	125849001	97850104

**58**	**Quercetin 3-*O*-(6"-pentosyl)-hexoside**	1104406117	1081853033	0	0	0	0	303060109	367910352	54181487	8247647
**59**	**Kaempferol 3-*O*-hexoside-7-*O*-hexuronide**	0	0	23660811	25186181	0	0	0	0	0	0
**60**	**Myricetin 3-*O*-pentoside**	0	0	0	0	56930056	46726202	0	0	0	0
**61**	**Quercetin 3-*O*-(6"rhamnosyl)-hexoside**	1069103935	1133430736	63794737	66943689	738019212	635446649	3032138991	3692714801	719878397	242843978
**62**	**Apigenin 6-*C*-hexoside**	0	0	13220097	19932297	0	0	0	0	11586296	12250224
**63**	**Patuletin 3-*O*-(6"rhamnosyl)-hexoside**	0	0	47729477	50343429	0	0	687912092	864012206	118042958	21250428
**64**	**Luteolin 7-*O*-(6"-pentosyl)-hexoside**	0	0	19398801	21851459	0	0	0	0	0	0
**65**	**Quercetin 3-*O*-hexoside**	3834896905	4068638776	3828930414	4164653695	5659662032	5514818994	1630085861	1791816428	4000273476	3446972206
**66**	**Quercetin 3-*O*-hexuronide**	0	0	66733645	70874485	60038089	79601806	883524204	1328860203	171660329	25744665
**67**	**Kaempferol 3-*O*-hexoside**	121726587	139137137	444348539	459185628	31671821	290520156	14946514	17959898	282703987	318863762
**68**	**Patuletin 7-*O*-**	0	0	0	0	0	0	991900186	1092544150	246817692	115611094

	**hexoside (Patulitrin)**										
**69**	**Patuletin 3-*O*-hexuronide**	0	0	0	0	0	0	1234849015	1827734423	2333383964	30470235
**70**	**Kaempferol 3-*O*-(6"-rhamnosyl)-hexoside**	42752539	46276448	21532803	23624838	353082029	255855842	160001656	191716463	0	0
**71**	**Luteolin 7-*O*-hexuronide**	58971420	58723631	1337505014	1442268248	44510479	660876879	40745528	47179391	59916081	47025819
**72**	**Quercetin 3-*O*-(6"-malonyl)-hexoside**	16852668	19051756	31235228	42262109	296411389	252318034	473076362	668030841	1258255793	914205891
**73**	**Quercetin 3-*O*-(6"-acetyl)-hexoside**	17688473	15466292	17887861	18107535	229549254	181593348	442290409	585041955	860520388	644142367
**74**	**Kaempferol 3-*O*-(6"-pentosyl)-hexoside**	112450868	114245881	13456101	13434580	0	0	38515188	42161396	0	0
**75**	**Quercetin 3-*O*-pentoside**	2951704770	3094863645	0	0	0	0	11118781	11855016	175047853	196732364
**76**	**Isorhamnetin 3-*O*-(6"-rhamnosyl)-hexoside**	0	0	0	0	0	0	116278434	123496486	6795675	696623
**77**	**Quercetin 3-*O*-rhamnoside**	7560433604	8000255725	158668407	164170730	1946948350	1492277661	163520382	134215241	0	0
**78**	**Isorhamnetin 3-*O*-hexuronide**	0	0	92938178	119518072	0	0	43180207	54804535	0	0

**79**	**6"-Malonyl-patulitrin**	0	0	0	0	0	0	467314410	541928771	66510940	10470129
**80**	**6"-Acetyl-patulitrin**	0	0	7492452	8217867	22770355	19183497	217139567	234282780	30618739	5301570
**81**	**Apigenin 7-*O*-hexuronide**	164773581	173190850	604739626	655850637	0	0	0	0	0	0
**82**	**Kaempferol 3-*O*-(6"-malonyl)-hexoside**	9631813	11172869	47539819	46631863	378630930	297048284	47588145	63647114	136101906	107134509
**83**	**Kaempferol 3-*O*-(6"-acetyl)-hexoside**	16325652	16002753	27186313	29409978	419170821	268125089	43726566	55668952	104524164	84017257
**84**	**Axillarin 7-*O*-hexoside (Axillaroside)**	0	0	0	0	0	0	70784822	65923100	0	0
**85**	**Quercetin 3-*O*-(6"-caffeoyl)-hexoside**	38347117	41526335	27988506	26981877	187657628	121018700	12463537	11383164	1327710630	1129072903
**86**	**Chrysoeriol 7-*O*-hexuronide**	0	0	757850162	813240992	0	0	39328045	52161547	0	0
**87**	**Chrysoeriol 7-*O*-hexoside**	72540723	76518921	273617382	292678601	145408423	201604432	687788786	1003471128	624565350	482033211
**88**	**Kaempferol 3-*O*-rhamnoside**	1357034275	1383433818	0	0	194193154	123872666	7489493	4484244	1699894	1476374
**89**	**Kaempferol 3-*O*-(6"-caffeoyl)-hexoside**	14101602	14218617	420605294	468308698	597053580	548954924	0	0	148119135	131199815

**90**	**Jaceosidin 7-*O*-hexuronide**	0	0	346620318	470906690	0	0	0	0	0	0
**91**	**Axillarin 3-*O*-(6"-malonyl)-hexoside**	0	0	0	0	0	0	55020570	77466429	0	0
**92**	**Apigenin 7-*O*-(6"-caffeoyl)-hexoside**	0	0	17412823	18537490	3549922	3407113	0	0	0	0
**93**	**Quercetin 3-*O*-(2"-caffeoyl)-pentoside**	19131340	19638574	0	0	0	0	0	0	0	0
**94**	**Methyl kaempferol 3-*O*-(6"-acetyl)-hexoside**	0	0	478551389	543859186	0	0	0	0	0	0
**95**	**Kaempferol 3-*O*-(2"-coumaroyl)-rhamnoside**	0	0	0	0	153655467	132156057	0	0	0	0
Flavonoid aglycones											
**96**	**Taxifolin**	0	0	41958511	47301422	0	0	0	0	5536629	4487697
**97**	**Eriodyctiol**	1500266	1771738	1217834229	1328186412	15023019	434159003	10368651	8751840	4600038	3865721
**98**	**Luteolin**	7395323	7049113	4820824604	5291276890	141642364	1958443081	81642673	48753635	229900171	214455404
**99**	**Patuletin**	0	0	0	0	0	0	31007503	21501304	13882365	13377555
**100**	**Quercetin**	193512970	299239321	89754049	86858346	367962756	270007336	229857440	201772558	32929362	13046815
**101**	**Isorhamnetin**	3158913	3022072	1440548914	1623009650	32624946	572630807	96008935	87517631	1235760398	1209370764

**102**	**Axillarin**	0	0	2633259093	2988232752	30313138	1035633015	17891660	14227845	16502423	15228656
**103**	**Naringenin**	2207088	2308793	137885519	156697319	26835762	70825427	32574881	29156148	6780695	3515462
**104**	**Apigenin**	1524493	1005802	2344558119	2587779144	23957957	833623500	150242372	140608927	122769580	107291888
**105**	**Kaempferol**	208206949	295288768	502129017	597409421	928737629	832874685	62786207	55331140	0	0
**106**	**Chrysoeriol**	0	0	1738432600	1879568494	637532525	659406365	315191977	296430146	1197263067	1122700021
**107**	**Jaceosidin**	2418225	3146119	1307807885	1449010358	21667195	566127096	0	0	122134199	112463717
**108**	**Eupatin**	0	0	607701788	646068706	0	0	0	0	93589649	91526028
**109**	**Tanetin**	0	0	412962745	457168629	3495311	75612765	771719	656306	0	0
**110**	**Acacetin**	0	0	4498779	5440446	4597613	3503823	225976371	219475340	26067416	3540144
**111**	**Cirsimaritin**	0	0	0	0	0	0	2600711379	2394158201	283920500	32639172
Terpenes											
**112**	**Argophyllin A**	1101025124	1057347173	0	0	0	0	0	0	0	0
**113**	**4α-Formyloxy-18-norgrindelic acid**	3114395593	3114767750	0	0	0	0	0	0	0	0
**114**	**Herbarumin I**	71499209	72125749	73874590	82653348	253795120	206625126	783096255	777780783	241430440	144612208
**115**	**Gibberellin A derivative 1**	857372072	771051107	0	0	0	0	0	0	0	0
**116**	**Gibberellin A derivative 2**	792197024	815863726	35528581	36586830	0	0	7005034	2866380	2248289	1119120
**117**	**2-β-hydroxy-6-**	2313217668	2335719493	0	0	0	0	0	0	0	0

	**deoxy-solidagolactone IV-18, 19-olide**										
**118**	**Viguilenin**	961477071	954674964	0	0	0	0	0	0	0	0
**119**	**Solidagenol isomer 1**	855168398	877961509	0	0	0	0	0	0	0	0
**120**	**Gibberellin A derivative 3**	1847551774	1751790492	0	0	0	0	0	0	0	0
**121**	**Solidagenol isomer 2**	223833126	242756033	0	0	7087742	13941746	0	0	0	0
**122**	**Solidagoic acid H**	7855204304	8849066812	0	0	0	0	0	0	0	0
**123**	**Solidagolactone VI**	6920948162	6733906887	0	0	0	0	0	0	0	0
Other metabolites											
**124**	**Benzyl pentosyl-hexoside***	432147983	425517320	86360279	97620690	449162401	337491394	360840563	465526160	819003670	552347053
**125**	**Tuberonic acid hexoside**	534822031	526505496	2080586398	2327518217	34409988	711091094	1794198350	2090392998	405274248	181957955
**126**	**6-Carboxyl-7-hydroxy-2,3-dimethylchromone**	0	0	0	0	1021329234	913447335	17915485	5001001	0	0
**127**	**Chrysophanol**	8977406	3365359	17934043	19452915	1149744	4993594	3710877	5546999	2169808	979842
**128**	**Hydroxy-octadecadienoic acid**	198438145	205421552	955918078	1102056855	1988365427	1874309420	1964798271	1817316731	1441539094	1289323256
**129**	**Octadecenedioic acid**	51658880	52325918	427023700	313484912	432134559	246334689	305708843	194179948	153489487	132126892

**130**	**Hydroxyoctadecatri-enoic acid**	211481601	240920115	983581210	1277263692	1277756865	1267682754	1563988498	1143394385	842330339	717101822

Table S3. Under-research plants from Asteraceae family and their main characteristics.

Plant	Voucher No.	Location of harvesting	Plant part	Label	Mean particle diameter (mm)	Moisture content (%)
Artemisia absinthium – Common wormwood	2-1574	Bački Petrovac 45°20'12"N; 19°40'12"E	Herb	A.a.H.	0.65	7.44±0.03
Carlina acanthifolia ssp. *utzka* – carline thistle	2-0032	Ozren (Sokobanja) 43°35'47"N; 21°53'50"E	Leaves	C.a.L.	0.62	8.33±0.06
			Roots	C.a.R.	0.28	10.75±0.19
Cichorium endivia – Curly endive	2-0100	Novi Sad 45°16'05"N; 19°48'13"E	Leaves	C.e.L.	0.38	7.70±0.06
Cichorium intybus – Chicory	2-0027	Bački Petrovac 45°20'12"N; 19°40'12"E	Herb	C.i.H.	0.42	8.19±0.04
			Roots	C.i.R.	0.58	7.75±0.02
Cota tinctoria – Golden marguerite	2-0038	Jezero (Sokobanja) 43°34'04"N; 21°54'11"E	Herb	C.t.H.	0.37	8.58±0.06
Eupatorium cannabinum – Hemp-agrimony	2-0034	Vrmdža (Sokobanja) 43°44'02"N; 21°49'41"E	Herb	E.c.H.	0.93	7.65±0.09
Helianthus tuberosus – Jerusalem artichoke	2-0099	Bački Petrovac 45°20'12"N; 19°40'12"E	Herb	H.t.H.	0.26	9.00±0.12
			Roots	H.t.R.	0.34	9.61±1.83
Inula ensifolia – Sword-leaf inula	2-0040	Sesalac (Sokobanja) 43°40'37"N 21°58'54"E	Herb	I.e.H.	0.26	9.31±0.05
Inula helenium – Elecampane	2-0028	Bački Petrovac 45°20'12"N; 19°40'12"E	Herb	I.h.H.	0.67	7.86±0.05
			Roots	I.h.R.	1.04	8.44±0.17
Inula oculus-christi – Christ's eye	2-0041	Šarbanovac (Sokobanja) 43°57'35"N; 22°04'22"E	Herb	I.o.H.	0.42	8.94±0.03
Lactuca serriola – Prickly lettuce or milk thistle	2-0098	Bački Petrovac 45°20'12"N; 19°40'12"E	Herb	L.s.H.	0.48	8.56±0.05
Silybum marianum – Milk thistle	2-0097	Bački Petrovac 45°20'12"N; 19°40'12"E	Seeds	S.m.S.	0.57	6.73±0.09
Solidago virgaurea – European goldenrod	2-0096	Bački Petrovac	Herb	S.v.H.	0.42	6.79±0.05

		45°20'12''N; 19°40'12''E				
Sonchus arvensis – Field sow thistle	2-0029	Bački Petrovac 45°20'12''N; 19°40'12''E	Herb	S.a.H.	0.42	8.43±0.05
Tanacetum macrophyllum – Big-leaf white tansy	2-0094	Kukavica (Vučje) 42°49'38''N; 21°55'32''E	Herb	T.m.H.	0.53	8.74±0.12
Tanacetum parthenium – Feverfew	2-0095	Kukavica (Vučje) 42°49'38''N; 21°55'32''E	Herb	Ta.par.H.	0.54	7.71±0.06
Tanacetum vulgare – Tansy	2-0033	Jezero (Sokobanja) 43°34'04''N; 21°54'11''E	Herb	T.v.H.	0.58	7.87±0.05
Tragopogon pratensis – Jack-go-to-bed-at-noon	2-0035	Novi Sad 45°16'05''N; 19°48'13''E	Herb	Tr.pra.H.	0.59	9.05±0.09
Tussilago farfara – Coltsfoot	2-0030	Vrelo (Sokobanja) 43°37'48''N; 21°59'31''E	Leaves	T.f.L.	1.05	7.43±0.17

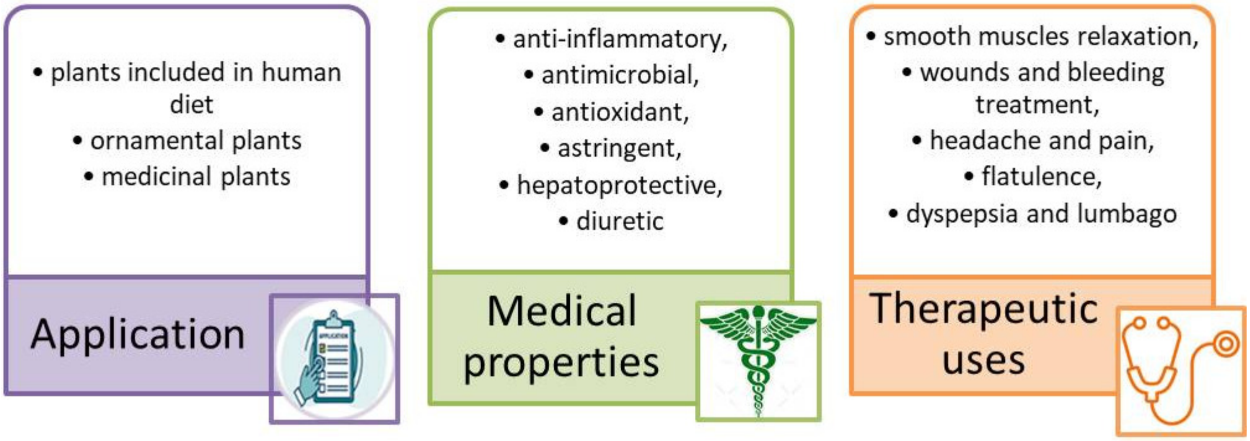

Figure S1. Application, medical properties and therapeutic use of plants from Asteraceae family.

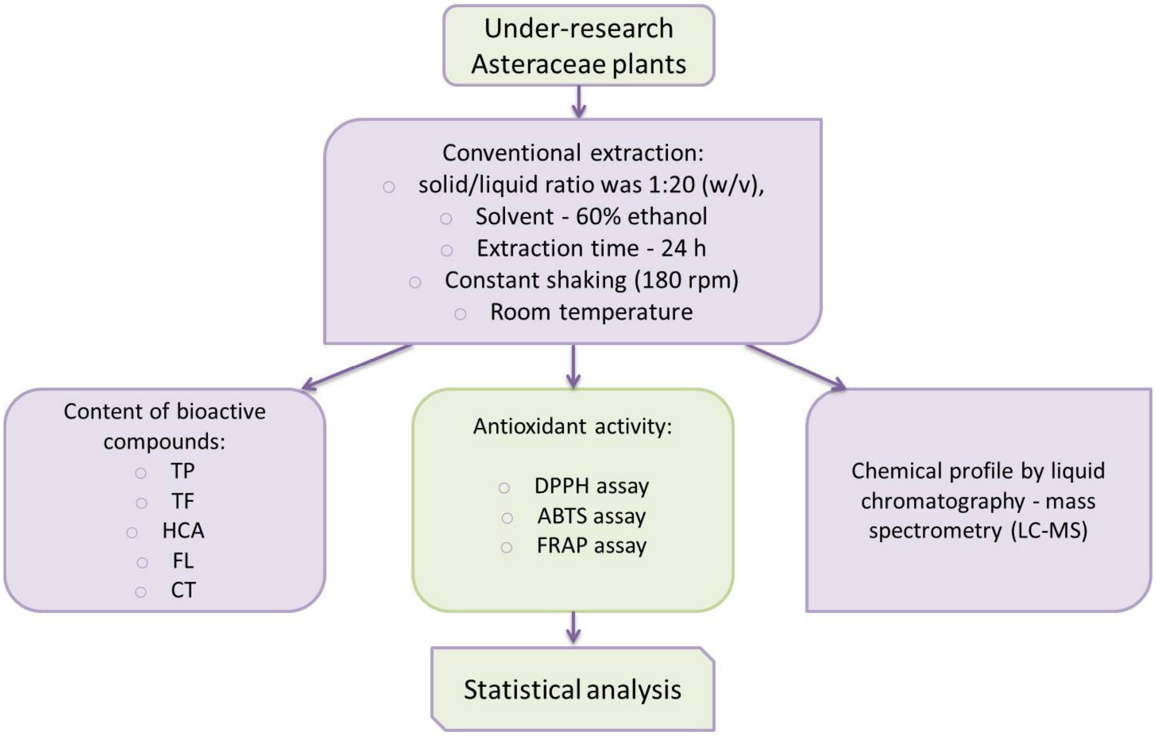

Figure S2. Experimental design for under-researched Asteraceae plants.

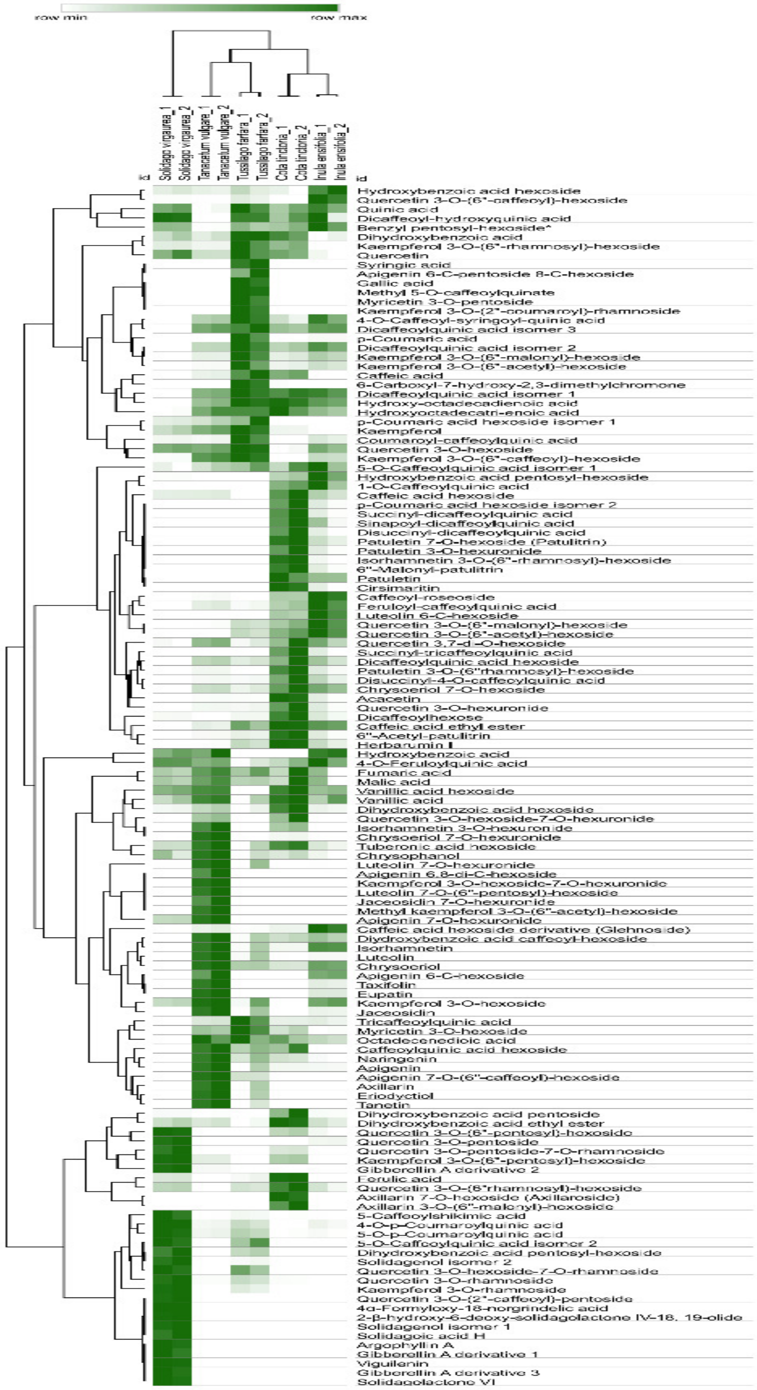

Figure S3. Heatmap of the scaled data of untargeted metabolite analysis, with the samples (both columns and rows) arranged according to the HCA (Spearman method of cluster agglomeration). Intensity of green color indicate the abundance of the compounds in samples.

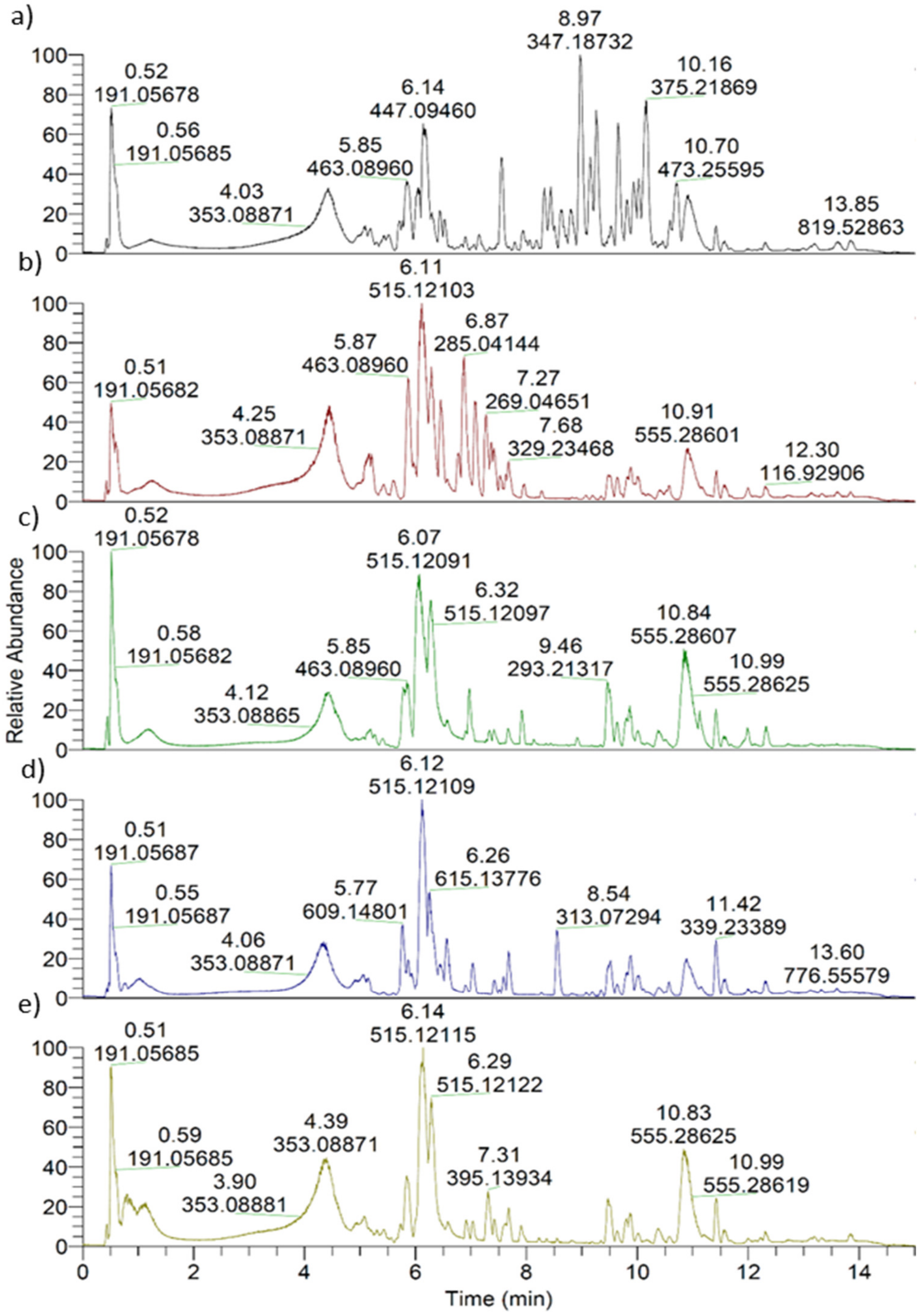

Figure S4. Base peak chromatograms for (a) *S. virgaurea* extract; (b) *T. vulgare* extract; (c) *T. farfara* extract; (d*) C. tinctoria* extract; and (e) *I. ensifolia* extract.
